# Solid State and Solution Study on the Formation of Inorganic Anion Complexes with a Series of Tetrazine-Based Ligands

**DOI:** 10.3390/molecules24122247

**Published:** 2019-06-16

**Authors:** Matteo Savastano, Celeste García-Gallarín, Claudia Giorgi, Paola Gratteri, Maria Dolores López de la Torre, Carla Bazzicalupi, Antonio Bianchi, Manuel Melguizo

**Affiliations:** 1 Department of Chemistry “Ugo Schiff”, University of Florence, Via della Lastruccia, 3-13, 50019 Sesto Fiorentino, Italy; matteo.savastano@unifi.it (M.S.); claudia.giorgi@unifi.it (C.G.); 2 Department of Inorganic and Organic Chemistry, University of Jaén, 23071 Jaén, Spain; cgarcia@ujaen.es (C.G.-G.); mdlopez@ujaen.es (M.D.L.d.l.T.); 3 Department of NEUROFARBA-Pharmaceutical and Nutraceutical Section, and Laboratory of Molecular Modeling Cheminformatics & QSAR, University of Florence, Via Ugo Schiff 6, 50019 Sesto Fiorentino, Italy; paola.gratteri@unifi.it

**Keywords:** *s*-tetrazines, anion complexes, anion–π interactions, lone pair–π interactions, H bonding, supramolecular forces, crystal structures, stability constants, complexation enthalpy

## Abstract

Four molecules (L1–L4) constituted by an s-tetrazine ring appended with two identical aliphatic chains of increasing length bearing terminal morpholine groups were studied as anion receptors in water. The basicity properties of these molecules were also investigated. Speciation of the anion complexes formed in solution and determination of their stability constants were performed by means of potentiometric (pH-metric) titrations, while further information was obtained by NMR and isothermal titration calorimetry (ITC) measurements. The crystal structures of two neutral ligands (L3, L4) and of their H_2_L3(ClO_4_)_2_∙2H_2_O, H_2_L4(ClO_4_)_2_∙2H_2_O, H_2_L3(PF_6_)_2_, and H_2_L3(PF_6_)_2_∙2H_2_O anion complexes were determined by single crystal X-ray diffraction. The formation of anion–π interactions is the leitmotiv of these complexes, both in solution and in the solid state, although hydrogen bonding and/or formation of salt-bridges can contribute to their stability. Evidence of the ability of these ligands to form anion–π interactions is given by the observation that even the neutral (not-protonated) molecules bind anions in water to form complexes of significant stability, including elusive OH^−^ anions.

## 1. Introduction

**Figure 1:**
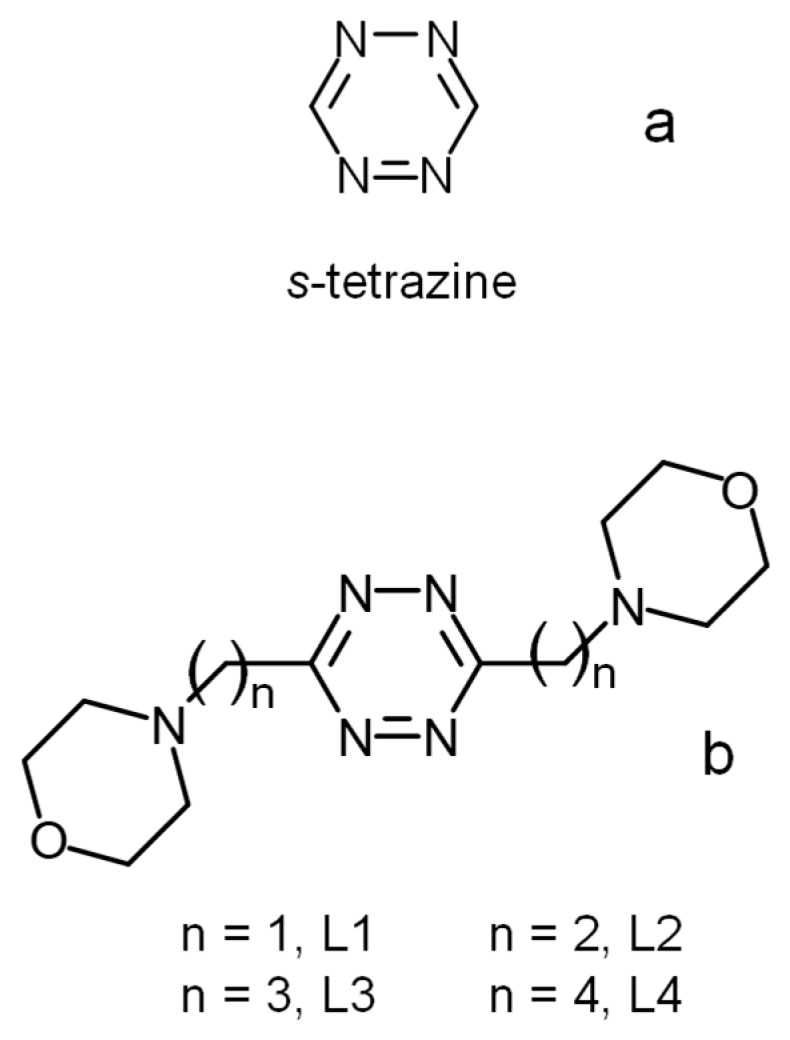
*s*-Tetrazine (**a**) and ligands L1–L4 (**b**).

*s*-Tetrazine (Figure 1a) and its derivatives (*s*-tetrazines) have aroused significant interest over the years because of their chemical and physico-chemical properties that make them appealing for various applications, from their early, but still continuing, use for the preparation of energetic materials (explosives, propellants, pyrotechnic compounds, etc.) to the preparation of active molecules for nonlinear optics, fluorescent devices, and electrochemical systems (organic batteries), to their involvement as structural elements and ancillary ligands in coordination compounds, or as functional groups for the assembling of supramolecular systems [1].

As for supramolecular chemistry, electronic features of s-tetrazine, such as a positive quadrupole moment, a high molecular polarizability, dispersion forces, or even electron sharing, have recently inspired the possible use of this heteroaromatic ring as a functional group to bind anions, an assumption that found solid foundations on ab initio calculations [2,3,4,5] and on the experimental evidence that anion–*s*-tetrazine ring interactions play an active role in determining the architecture of some metalla-macrocycles [6,7]. More recently, it was shown that *s*-tetrazine rings do behave as a binding site for anions even in solution [8,9,10,11,12], the anion–*s*-tetrazine interaction emerging as a promising tool for the construction of anion receptors as well as for the self-assembling of anion coordination frameworks (ACF) and polymers (ACP) [13,14].

In particular, we recently found that ligands L1 and L2 (Figure 1b) form complexes with inorganic anions even in water, a solvent that opposes the formation of anion complexes by virtue of its high dielectric constant and high ability to solvate anions and form competitive hydrogen bonds. In the crystal structures of the diprotonated forms of L1 and L2 (H_2_L1^2+^, H_2_L2^2+^), the inorganic anions are invariably located over the positive electrostatic potential of the ligands’ tetrazine ring. Despite the different sizes and shapes, the anions are always at short interaction distances, and, notably, the two ammonium groups in the ligands are involved in only some cases in salt-bridge interactions. In solution, both charged and uncharged ligand forms bind anions with moderate, but significant, stability. Trends of thermodynamic data were consistent with a prominent contribution of such anion–π interactions even in solution [8,9,10,11].

In previous studies dealing with the formation of anion complexes with a polyamine ligand containing an electron-deficient nitroso-amino-pyrimidine group, we showed that linear correlations between the binding free energies and the receptor charge for systems combining salt-bridge and anion–π interactions may offer the opportunity to determine the individual contribution of each type of interaction. In the specific case, the free energy change derived for the formation of anion–π interactions with such anions as SO_4_^2−^, SeO_4_^2−^, S_2_O_3_^2−^, and Co(CN)_6_^3−^ in water was in the range −8.6 to −12.0 kJ/mol [15,16,17]. The free energy changes for the interaction of L1 and L2 free ligands (not protonated) with inorganic anions (ClO_4_^−^, PF_6_^−^, SO_4_^2−^) fall within this range, in agreement with a principal contribution to complex stability deriving from anion–π interactions.

In the case of organic anions, the stabilization of complexes with L1 and L2 appears to be regulated by a more subtle interplay of different contributions, including additional supramolecular forces, such as π–π stacking interactions, host–guest size matching, stereochemistry of the interacting groups, and the hydrophobic effect, that offers the possibility to finely tune the anion–ligand interaction [18].

In the present paper, we present the binding ability toward inorganic anions of the two new ligands L3 and L4 (Figure 1), which are larger homologues of L1 and L2, with the aim of obtaining a better understanding of the roles played by anion–π contributions in the formation of complexes with *s*-tetrazines. In L3 and L4, there is a larger distance (with respect to L1 and L2) between the tetrazine ring, which is the location of anion–π interactions, and the morpholine groups that, upon protonation, become available for the formation of salt-bridges, which furnishes a structural element for a better separation of the two contributions or for the exclusion of one of them. In the work described hereafter, we analyze the anion binding properties of L3 and L4 according to X-ray diffraction (XRD) studies in the solid state and thermodynamic (potentiometric, isothermal titration calorimetry (ITC)) and ^1^H-NMR analysis of the complex systems in aqueous solution.

## 2. Results and Discussion

### 2.1. Crystal Structure of L3 and L4 Free Ligands

**Figure 2:**
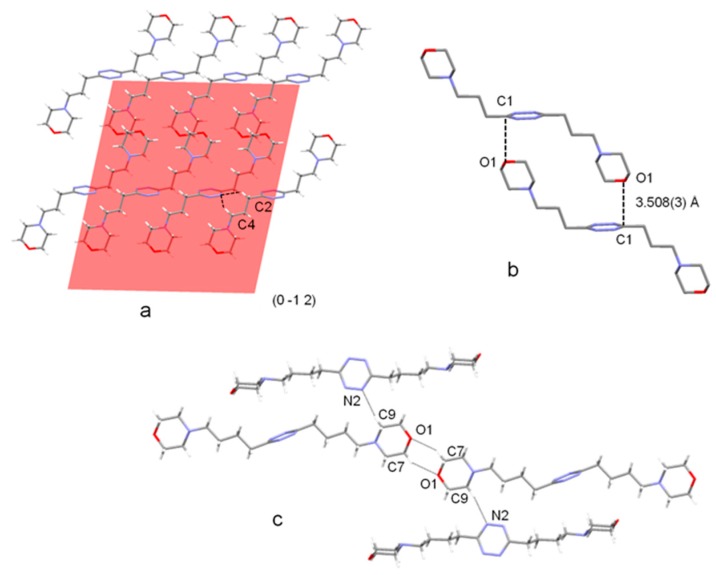
(**a**) (0 -1 2) Plane in the crystal packing of L3 showing the chair-type conformation of L3 and the CH∙∙∙N contacts between parallel molecules; (**b**) infinite net ribbons formed by ligand molecules of adjacent planes in the (0 -1 2) family; (**c**) chair-type conformation of L4 in the crystal structure of the free ligand and infinite chains formed by head-to-tail connected molecules. Selected hydrogen bond contacts are shown.

In the crystal structures of the free ligands, L3 and L4 molecules assume centrosymmetric chair-type conformations with all trans torsion angles in their propylenic (L3) and butylenic (L4) chains (Figure 2a,c).

The crystal packing of free L3 can be properly described considering the (0 -1 2) family of planes. Each of these planes (Figure 2a) contains parallel L3 molecules interacting via C-H∙∙∙N hydrogen bonds, which are the shortest intermolecular contacts in the crystal and involve tetrazine nitrogen and propylenic carbon atoms (C4∙∙∙N1 3.508(3) Å, C2∙∙∙N1 3.566(4) Å). Pairs of ligands belonging to adjacent (0 -1 2) planes are strongly connected by two symmetric contacts between the morpholine oxygen and the C1 tetrazine carbon atoms (O1∙∙∙C1 3.508(3) Å, Figure 2b), giving rise to infinite net ribbons growing parallel to the (1 1 -1) crystallographic plane.

In contrast to the behaviour just described for L3, and previously reported for L1 and L2 [6], the tetrazine ring of L4 does not show any tendency to interact with lone pairs of neutral donor atoms in its crystal structure as free ligand. The principal interactions stabilizing the crystal packing of L4 are couples of symmetric O1∙∙∙H-C7 hydrogen bonds (C∙∙∙O 3.471(2) Å) linking L4 molecules through their morpholine units, in a head-to-tail fashion, to form infinite chains (Figure 2c). These chains are then connected by C-H∙∙∙N interactions, similar to those found in the L3 structure, involving tetrazine ring and morpholine units from different chains (C9∙∙∙N2 3.443(2) Å).

### 2.2. Crystal Structure of H_2_L3(ClO_4_)_2_∙2H_2_O

**Figure 3:**
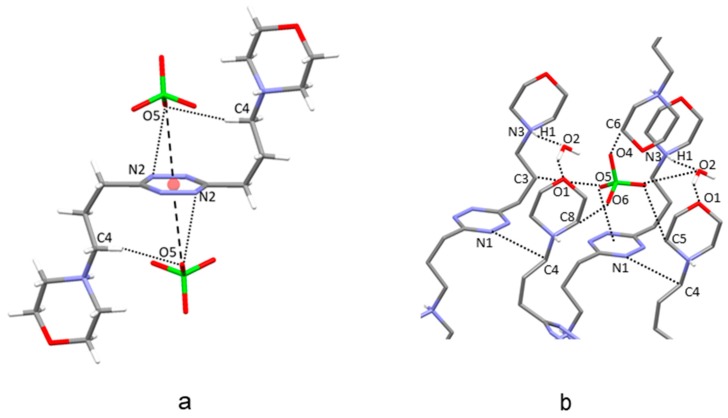
(**a**) Chair-type conformation of the ligand in the crystal structure of H_2_L3(ClO_4_)_2_∙2H_2_O; anion–π interactions with the symmetry-related ClO_4_^−^ anions. (**b**) Interactions established by ClO_4_^−^ in the pocket formed by the symmetry-related ligands and the cocrystallized water molecules.

The most important feature of this ClO_4_^−^ complex is the presence of two symmetry-related anions forming anion–π interactions with the tetrazine ring of L3 (Figure 3a). Each perchlorate anion places one of its oxygen atom at 3.12 Å from the ring centroid, shifted toward the N2 nitrogen (O5∙∙∙N2 3.049(8) Å, offset from the ring centroid 0.84 Å). L3 assumes a symmetric chair-type conformation similar to that shown in the free ligand structure, but with C–C propylenic bonds adopting a cis-trans sequence. This conformation allows for the carbon atom bearing the morpholine group to get in contact with the anion via a CH∙∙∙O interaction (C4∙∙∙O5 3.425(9) Å). Overall, each anion is placed in a pocket formed by the morpholine groups of symmetry-related L3 molecules, cocrystallized water molecules, and the tetrazine ring involved in the anion–π interaction. Figure 3b shows the molecules defining this pocket along with the shortest ligand–anion and ligand–ligand contacts (contact distances are in Table S1). Interestingly, as already found for several crystal structures of the anion complexes of this series of ligands, the protonated morpholine nitrogen prefers to interact with the cocrystallized water (N3-H1∙∙∙O2 1.98(7) Å) rather than to form hydrogen bonds with the coordinated ClO_4_^−^ anions.

### 2.3. Crystal Structure of H_2_L4(ClO_4_)_2_∙2H_2_O

**Figure 4:**
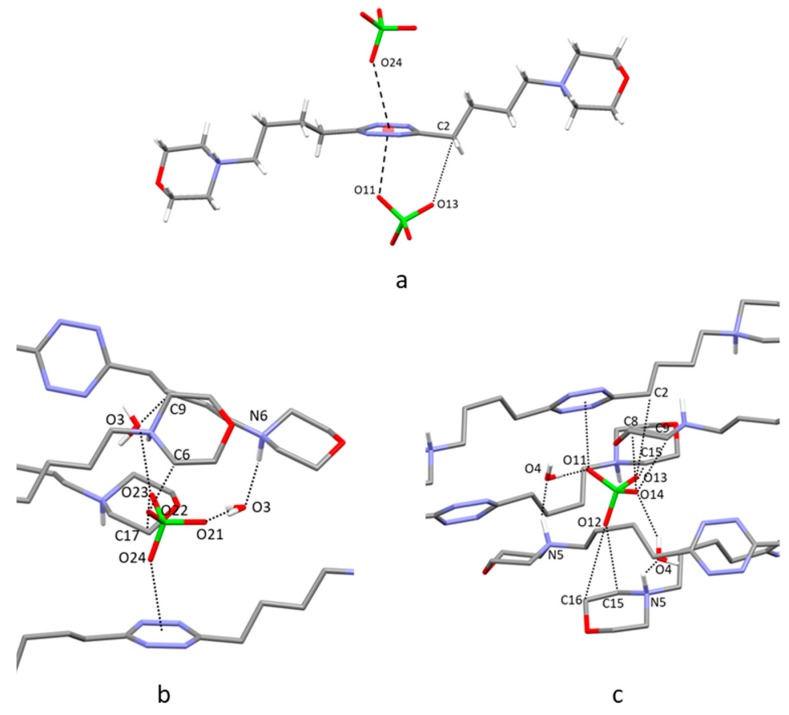
(**a**) Anion–π interactions in the crystal structure of H_2_L4(ClO_4_)_2_∙2H_2_O. (**b**,**c**) Interactions established by ClO_4_^−^ ions in the pockets formed by the symmetry-related ligands and the cocrystallized water molecules.

In the crystal structure of the ClO_4_^−^ complex of L4, the ligand adopts an open conformation with one butylenic arm forming all trans C–C torsion angles and one giving rise to trans-cis-trans sequences (Figure 4a). Anion–π interactions involve the tetrazine above and below the ring in a manner similar to that seen in the crystal structure of H_2_L3(ClO_4_)_2_∙2H_2_O. Each perchlorate anion places one oxygen atom at close range of the heteroaromatic ring, 2.85 Å (O11) and 3.12 Å (O24) from the ring centroid with offset values of 0.34 Å (O11) and 0.86 Å (O24). The only additional relevant contact is established between the O13 oxygen atom and one of the carbon atoms of the butylenic chain with all trans C–C bonds (O13∙∙∙C2 3.387(5) Å). Despite the different ligand conformation, the crystal packing of H_2_L4(ClO_4_)_2_∙2H_2_O shows interesting similarity to the perchlorate complex of L3, as the anions are again lodged in pockets defined by the tetrazine ring, the aliphatic pendant arms, and the cocrystallized water molecules, which bridge the protonated morpholine nitrogen atoms and the perchlorate anion inside the pocket (Figure 4b,c and Table S2).

### 2.4. Crystal Structure of H_2_L3(PF_6_)_2_

**Figure 5:**
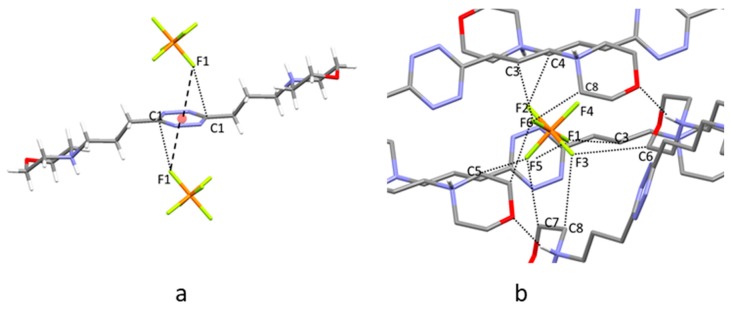
(**a**) Anion–π interactions in the crystal structure of H_2_L3(PF_6_)_2_. (**b**) Interactions established by the PF_6_^−^ ions in the pocket formed by the symmetry-related ligands.

In this crystal structure, L3 assumes a centrosymmetric open conformation with its propylenic chains characterized by all trans torsion angles (Figure 5a). Two symmetry-related PF_6_^−^ anions form anion–π interactions with the tetrazine ring, as already found for ClO_4_^−^ in H_2_L3(ClO_4_)_2_∙2H_2_O. Each PF_6_^−^ anion is affected by a slight rotational disorder: two anions were found to share almost the same position with very similar occupancy factors (0.46/0.54 for anion A and B, respectively). For the sake of simplicity, we discuss here only the intermolecular contacts given by the most representative PF_6_^−^ anion B, while a list of contacts referring to both PF_6_^−^ anions has been included in the ESI (Table S3). The fluorine atom closest to the tetrazine ring lies 2.89 Å from the ring centroid, slightly moved toward the C1 carbon atom (Figure 5a, F1∙∙∙C1 3.049(8) Å, offset from the ring centroid 0.59 Å). Each anion is positioned inside a pocket similar to those found in the previous structures, where it forms several CH∙∙∙F contacts with the pendant arms from symmetry-related molecules (Figure 5b, Table S3). Once again, the protonated morpholine nitrogen atoms prefer to form hydrogen bonds with morpholine oxygen atoms of adjacent ligand molecules rather than with the coordinated PF_6_^−^ ions.

### 2.5. Crystal Structure of H_2_L3(PF_6_)_2_∙2H_2_O

**Figure 6:**
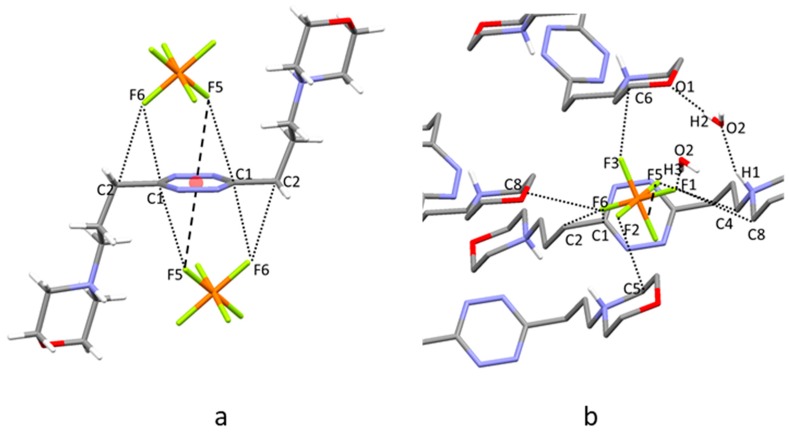
(**a**) Anion–π interactions in the crystal structure of H_2_L3(PF_6_)_2_∙2H_2_O. (**b**) Interactions established by the PF_6_^−^ ions in the pocket formed by the symmetry-related ligands.

The ligand assumes a centrosymmetric chair-type conformation and involves its tetrazine ring in two anion–π interactions with two symmetry-related PF_6_^−^ anions, analogously to what has already been found for H_2_L3(ClO_4_)_2_∙2H_2_O. The closest contact between PF_6_^−^ and the tetrazine ring is formed by F5, lying 3.03 Å from the ring centroid, slightly displaced toward the C1 carbon atom (Figure 6a, F5∙∙∙C1 3.135(5) Å, offset from the ring centroid 0.43 Å), but additional interesting contacts are given by F6 with C1 and C2 carbon atoms (2.988(5) Å and 3.125(6) Å, respectively). As for the previous structures, several CH∙∙∙F contacts stabilize the anion inside a pocket formed by the pendant arms of symmetry-related molecules, cocrystallized water molecules, and the tetrazine ring involved in the anion–π interaction (Figure 6b). Also, in this case, the protonated morpholine nitrogen atoms prefer to form hydrogen bonds with morpholine oxygen atoms of contiguous ligand molecules rather than with the PF_6_^−^ ions (Table S4). Further contacts are formed by the water molecules that bridge symmetry-related morpholine groups (N-H∙∙∙O-H∙∙∙O donor-acceptor distances 2.798(4)–2.794(5) Å) and interact with the coordinated PF_6_^−^ anions (O2∙∙∙F1 2.948(4) Å).

### 2.6. Analysis of the Crystal Structures of Anion Complexes

The interaction of anions with electron-deficient arenes can take place above the plane of the π system according to both centered and off-center interaction geometries. In the latter case, the anion is placed over the periphery of the ring and charge transfer (CT) complexes can be formed as a result of some covalent character of the interaction with ring atoms [19].

Conversely, in the former case, the anion lies above the centroid of the ring, where the CT contribution to the anion–π interaction is expected to be negligible. DFT calculations performed for the diprotonated L1 and L2 ligands showed that the lowest unoccupied molecular orbital (LUMO) of these molecules is localized on the tetrazine ring, which might accept an electronic charge from the coordinated anions. Nevertheless, a natural population analysis indicated that the CT contribution is modest in the L1 and L2 anion complexes [8,9].

**Figure 7:**
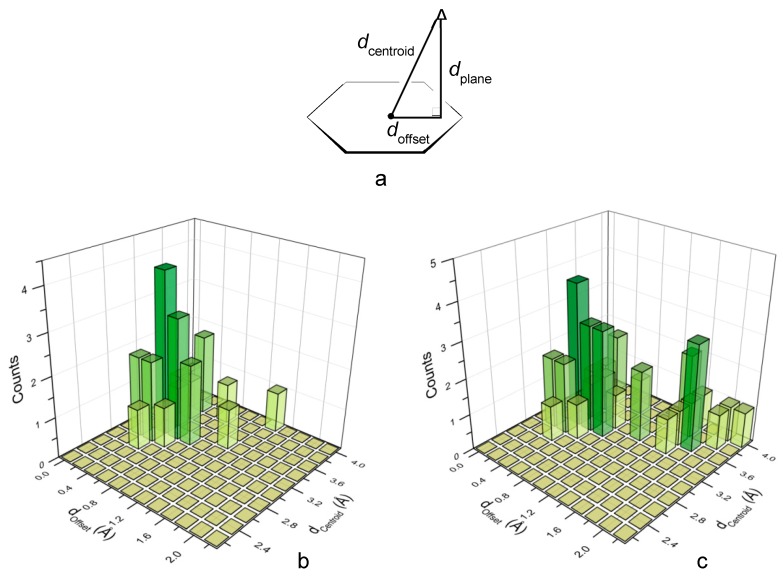
(**a**) Displacement of an atom A from the center (centroid) of the tetrazine ring. (**b**) Frequency of shorter anion–ring contacts, for each anion, in the d_centroid_ versus d_offset_ plane. (**c**) Frequency of all anion–ring contacts in the d_centroid_ versus d_offset_ plane. Data from crystal structures of H_2_L1^2+^ with PF_6_^−^, ClO_4_^−^; H_2_L2^2+^ with HF_2_^−^, Cl^−^, Br^−^, I^−^, I_3_^−^, SCN^−^, NO_3_^−^, PF_6_^−^, ClO_4_^−^; H_2_L3^2+^ with PF6^−^, ClO_4_^−^; H_2_L4^2+^ with PF_6_^−^, ClO_4_^−^ [8,9,10,11].

Indeed, an analysis of all crystal structures of L1–L4 complexes with inorganic anions (those reported in this paper and those previously published [8,9,10,11]) clearly evidences the preference of these ligands for the centered interaction mode. The location of the anions above the tetrazine ring can be described by the d_offset_, d_centroid_, and d_plane_ parameters defined in Figure 7a. The d_offset_ has a value of 0 Å for a perfectly centered anion and a value of 1.4 Å when the anion is exactly located above a ring atom, assuming a benzene model for the *s*-tetrazine ring. When the shortest anion-centroid distances (d_centroid_) in each crystal structure are taken into account, the corresponding d_offset_ values gather in proximity of the centroid (Figure 7b), accounting for the anticipated preference of L1–L4 for the centred interaction. Nevertheless, due to the polyatomic nature of several anions, further longer d_centroid_ values can be found between the same anion and the same tetrazine ring. Figure 7c shows the frequencies of anion–ring contacts when a d_offset_ threshold of 4 Å is adopted. The consideration of the longer d_offset_ values, although possibly leading to biased information on the preferred interaction mode, provides a more complete picture of the anion–ring interaction. Thus, using longer offset values may bring out the existence of weaker, yet favorable, binding contributions. Analogous information is obtained when the structural analysis is conducted by considering the anion distance from the tetrazine plane (Figures S9 and S10). In particular, Figure S10 shows that, for anions (or atoms of polyatomic anions) closer to the ring plane, the d_centroid_ and d_plane_ parameters tend to be very similar, that is, for the shortest contacts the interacting atoms tend to be positioned exactly above the centroid.

### 2.7. Ligand Protonation in Solution

The determination of ligand protonation constants is preliminary to the determination of the stability constants of an anion complex and performed by means of potentiometric (pH-metric) titrations, since ligand basicity is enhanced by interaction with the anions, a basilar property for the application of this method. Protonation constants of L3 and L4, determined in 0.10 M Me_4_NCl aqueous solution at 298.1 ± 0.1 K, are listed in Table 1, along with the relevant enthalpy changes (Δ*H*°), determined by ITC, and the derived entropic contributions (*T*Δ*S*°). The analogous data previously determined [5] for L1 and L2 have been included in the same table. An inspection of protonation constants (first and second protonation steps) evidences that the morpholine groups undergo a progressive basicity enhancement from L1 to L4, with a very large increase from L1 to L2 followed by an attenuated growth (L1 <<< L2 << L3 <L4) featuring an asymptotic approach to a limit value (Figure S11). Interestingly, the basicity of L1, at both the first and second protonation stages, relies on a largely favorable entropic contribution. The entropic contribution decreases markedly for longer L2–L4 ligands, becoming unfavorable in the second protonation stages of L3 and L4. Conversely, the enthalpic contribution, which is small for L1, becomes pretty favorable for L2 and definitely favorable for L3 and L4.

This peculiar behaviour of thermodynamic parameters for protonation of L1–L4 is not what is expected from a series of terminal diamines of increasing length, which should display a smooth increase in basicity, with increasing separation of the amine groups accompanied by largely favorable and dominant enthalpy changes, smoothly increasing with chain length, and by minor entropy contributions [20]. Although inductive effects due to the electron deficiency of the tetrazine ring works in the correct direction (the electron density, hence the basicity, of the morpholine nitrogen is decreasingly detracted as the aliphatic connection with the tetrazine ring becomes longer), we cannot expect that they are completely responsible for the large increase in basicity of the morpholine groups observed along the L1–L4 series [20]. Peculiar solvation properties of these molecules are seemingly making a significant contribution to their basicity behaviour. They are a consequence of the dissimilar chemical features of the two main components that participate in the ligand structure (namely, the aromatic *s*-tetrazine nucleus and the pendant morpholine rings) and of how their cooperative interaction to bind to water molecules can modify their solvation behaviour in aqueous media. Regarding the tetrazine ring, a molecular dynamics simulation of the hydration of *s*-tetrazine, performed with explicit treatment of solvent molecules, showed that *s*-tetrazine is poorly solvated in a water cave with weak bonding to its hydrogen atoms and occasional short-lived hydrogen bonds to the nitrogen atoms. Structures with the water molecule directly interacting with the π-system of *s*-tetrazine were found to be significantly weaker [21].

On the other hand, the solvation of the morpholine moieties is expected to obey to a completely different model characterized by the formation of H-bonds between water molecules (as H-donors) and the heteroatom basic centers of the morpholine ring [22], especially its nitrogen atom (N_morph_), which is anticipated to be the most basic center in the ligands.

**Figure 8:**
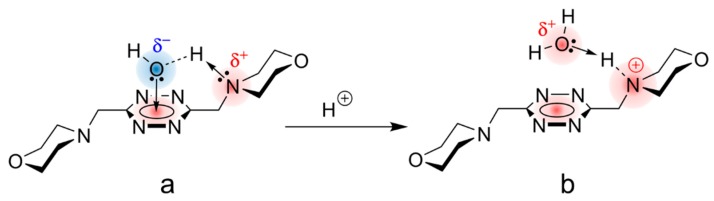
(**a**) Ligand–water adduct sustained by a cooperative hydrogen bond and lone pair–π interactions. (**b**) Disassembling of the ligand–water adduct after protonation.

In the case of L1 and L2, our molecular modeling calculations show (Figures S12–S19) that direct interaction of a water molecule with the π-system of *s*-tetrazine becomes favorable even thanks to the participation of hydrogen bonding with the nitrogen atom of morpholine groups in the ligand arms. It must be taken into account that such H-bonding not only results in a favorable spatial arrangement for the water molecule to interact with both the N_morph_ and the *s*-tetrazine ring, but also produces a strong polarization of the H-O_w_ bond in the water molecule involved, so that the electron density on the oxygen atom (O_w_) will experience an appreciable increase that benefits its interaction with the π-acidic center of the *s*-tetrazine aromatic ring (Figure 8).

A similar binding mode was also found for the interaction of alcoholic oxygen with perfluorinated aromatic groups in organic solvents [23]. As for L3 and L4, the arms are exceedingly long to accomplish the same function, the morpholine groups move further and further away from the tetrazine rings, and the preferred interaction mode of the water molecule returns to be parallel to this ring, in the case of L3 (Figure S14), while it involves hydrogen bonding to the morpholine nitrogen, which is located far from the tetrazine, in the case of L4 (Figure S15).

Then, for L3 and L4, a simple model based on solvation of rather independent moieties (tetrazine and morpholines) subjected to weak mutual interaction could be acceptable, whereas for L1 and L2 the adduct generated by binding of a ligand molecule with a water molecule through a lone pair–π interaction between the O_w_ and the tetrazine ring, assisted by an H-bonding HO_w_···H···N_morph_, should be considered to be the core around which to build up the solvation shell. Also, in such water–ligand adducts, the strong polarization of the water molecule taking part in the adduct will determine a stricter orientation and tighter binding of solvent (water) molecules around the *s*-tetrazine nucleus compared with the looser solvation expected for the tetrazine residue in L3 and L4 (see above).

These two different solvation arrangements in aqueous media help to explain the diverse thermodynamic behaviour observed in the protonation processes of the ligand molecules L1–L4. The thermodynamic data of ligands’ protonation (Table 1) show that for L1, and at lower extent for L2, a rearrangement of the water cage around the tetrazine ring, involving disruption of the special cohesion of water molecules around the cavity, takes place according to a process that is expected to be endothermic and exoentropic. This process is triggered by protonation of the N_morph_ that transforms this originally basic center into an acidic one that is unable to participate as an H-acceptor in the cooperative H-bond that sustains the structure of the water–ligand adduct (Figure 8). Consequently, the adduct will disassemble after protonation, breaking the previous orientation of the solvent molecules around the tetrazine nucleus. Then, the low Δ*G*° (low basicity), the poorly favorable (or unfavorable) Δ*H*°, and the largely favorable *T*Δ*S*° terms for the protonation of L1 would be a consequence of this phenomenon. Consistently, as the morpholine nitrogen atoms are located further and further away from the tetrazine ring, this phenomenon has a lower effect: L2 is still somewhat affected, while L3 and L4 display a normal protonation behaviour.

### 2.8. Anion Binding in Solution

The stability constants of the anion complexes formed by L1–L4 are listed in Table 1. Since these measurements were performed in the presence of 0.10 M Me_4_NCl, we must assume that all the equilibrium constants in this table are potentially affected by the competitive ligand interaction with Cl^−^. Analysis, by means of the computer program HYPERQUAD [24], of potentiometric (pH-metric) titrations performed with various ligand to anion molar ratios invariably showed the formation of 1:1 complexes, in contrast to the observed solid state (crystal structures) formation of 1:2 ligand to anion complexes, which, however, are favored by the requirement of charge neutrality in the presence of diprotonated ligand molecules. Unfortunately, we were not able to determine the stability constants of PF_6_^−^ complexes with L4 due to solubility problems (see the experiment section).

It was previously observed for L1 and L2 that the stability of the anion complexes increases slightly with ligand charge, a behaviour that was explained by assuming that electrostatic interactions are not the principal forces at stake [8,9,10,11]. In the case of L3 and L4, this behaviour becomes extreme, to the point that the stability constants of their complexes with ClO_4_^−^ and PF_6_^−^ are equal, within experimental errors, regardless of the ligand charge. That is, as the protonated morpholine groups are moved further and further away from the tetrazine ring, the effect of charge-charge attraction becomes ineffective. This observation reinforces the belief that anion–π interactions are of importance in stabilizing anion complexes with L1–L4 ligands. Nevertheless, some contribution from hydrogen bonding of the anions with protons of the ligand chains can be expected. Indeed, ^1^H-NMR spectra recorded at increasing ligand/anion ratios, in pure water, show that signals of chain protons sense anion complexation (Figures S20–S23), L3 appearing to be more involved than L4 in the interaction with the anions. However, in a medium of high Cl^−^ concentration (0.10 M), such as the one we adopted for our measurements, the ability of the ligand chains to form hydrogen bonds with ClO_4_^−^ and PF_6_^−^ is lower than in pure water due to saturation with Cl^−^. As a matter of fact, the free energy changes (−12.0 to −13.7 kJ/mol) for ClO_4_^−^ and PF_6_^−^ binding by L3 (protonated and not-protonated) fall near, or slightly above, the upper limit (−12.0 kJ/mol) previously found for anion–π interactions in water [12,13,14], suggesting that some hydrogen bonding of the anion with chain protons is still active, while the free energy changes (−6.3 to −9.1 kJ/mol) for ClO_4_^−^ binding by L4 (protonated and not-protonated) are close to the lower limit (−8.6 kJ/mol) in agreement with the loss of such a hydrogen bonding contribution.

The most salient property of these L1–L4 ligands is probably the ability of their neutral (not-protonated) forms to form stable complexes with hydroxide (OH^−^) anions in water. Actually, as far as we know, these are the first reported cases of non-covalent binding of hydroxide anions in water with metal-free synthetic receptors, even if examples of OH^−^ anions interacting with electron-poor aromatic groups in the solid state can be found in published crystal structures [25,26,27,28,29,30,31,32,33,34,35,36,37,38,39]. In only a few of them, the existence of anion–π interactions was noted [31]. As can be seen from Table 1, OH^−^ complexes with L3 and L4 are the most stable anion complexes formed by these ligands, independently of their protonation state. While the free energy changes of complexation do not show a particular trend for hydroxo-complexes [(L)OH]^−^ (L = L1–L4), the enthalpy changes (ΔH°) are considerably less exothermic (less favorable) for L3 and L4 than for L1 and L2, whereas the associated entropy changes (TΔS°), that are unfavorable (negative) for L1 and L2, become favorable (positive) for L3 and L4 (Table 1). Such behaviour might be indicative of a lower (or absent) participation of the ligand chains in stabilizing the OH^−^ complexes via hydrogen bonding, that is, the associated favorable enthalpic contribution for hydrogen bonding would be lost, while a favorable entropic contribution would be gained for the increased freedom of the not-coordinated ligand chains.

## 3. Materials and Methods

### 3.1. Materials

All reagents and solvents were of reagent-grade purity or higher. They were purchased from commercial sources and used without further purification unless otherwise stated. The anions used for potentiometric measurements were obtained as high-purity sodium salts from commercial sources and were used without further purification. Crystals of L3 and L4 suitable for X-ray analysis were prepared by slow evaporation at room temperature of chloroform solutions. Crystals suitable for X-ray analysis of H_2_L3(ClO_4_)_2_∙2H_2_O and H_2_L4(ClO_4_)_2_∙2H_2_O were obtained upon slow evaporation at room temperature of aqueous solutions of the ligands containing 3 equivalents of HClO_4_. Crystals of H_2_L3(PF_6_)_2_ and H_2_L3(PF_6_)_2_∙2H_2_O suitable for X-ray analysis were prepared by slow evaporation at room temperature of ligand solutions containing a fivefold excess of PF_6_^−^, added as KPF_6_, after acidification (pH 4) with HCl. ^1^H, ^13^C-NMR, FTIR (ATR), and HRMS (Q-TOF/ESI) spectra of L1 and L4 are reported in the Electronic Supporting Information (ESI, Figures S1–S8).

**Scheme 1:**
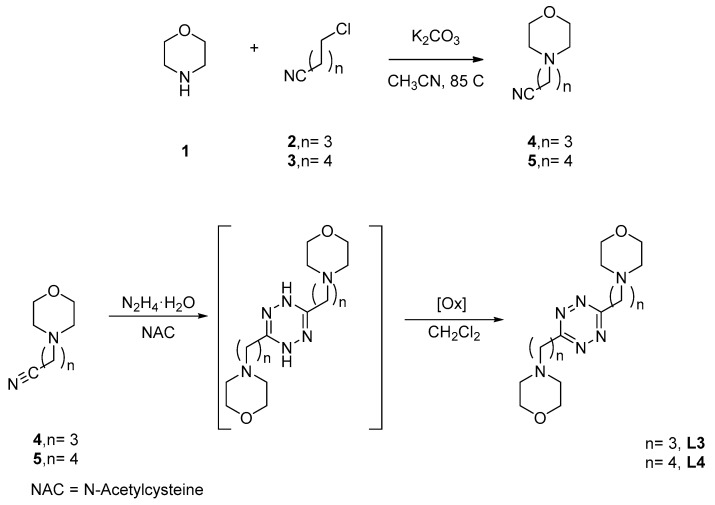
Synthesis of L3 and L4 ligands.

Synthesis of L3 and L4. The synthesis of ligands L3 and L4 was accomplished following a Pinner synthetic scheme, as in the case of previous 3,6-di(morpholin-4-y)alkyl-*s*-tetrazines [8], but in the absence of solvent (Scheme 1). Thus, the ω-(morpholin-4-yl)nitrile precursors were reacted with excess hydrazine hydrate in the presence of N-acetylcysteine as a catalyst at room temperature for three days to give the corresponding di(morpholin-4-y)alkyl-dihydro-*s*-tetrazine intermediates, which, after oxidation with atmospheric oxygen at room temperature, afforded the fully aromatic tetrazine derivatives L3 and L4 in excellent yields.

### 3.2. Preparation of ω-(morpholin-4-yl)nitrile Precursors

To a mixture of morpholine, **1**, (6.90 mL, 80 mmol) and anhydrous potassium carbonate (5.58 g, 40 mmol) in acetonitrile (50 mL), 4-chlorobutanenitrile, **2**, (1.94 mL, 20 mmol) or 5-chloropentanenitrile, **3**, (2.20 mL, 20 mmol) was added. The mixture was stirred for 72 h at room temperature. After that time, the crude was evaporated to dryness. Then, water (50 mL) was added and the mixture extracted with dichloromethane (3 × 30 mL). The organic extract was subsequently washed with water, dried over anhydrous Na_2_SO_4_, evaporated to dryness in a rotary evaporator, and, finally, purified by vacuum distillation to yield a yellowish oil that was identified as 3-(morpholin-4-yl)-butanenitrile (4) or 3-(morpholin-4-yl)-pentanenitrile (5), respectively [40,41]. Compound **4** was obtained in a 99% yield. ^1^H-NMR (CDCl_3_, 400 MHz): δ 3.67–3.65 (m, 4 H), 2.40–2.32 (m, 8H), 1.79 (quin, *J* = 6.7 Hz, 2H). ^13^C-NMR (CDCl_3_, 100 MHz): δ 119.8, 67.0, 56.8, 53.6, 22.5, 14.9. Compound **5** was obtained in a 95% yield. ^1^H-NMR (CDCl_3_, 400 MHz): δ 3.67–3.65 (m, 4H), 2.40–2.32 (m, 8H), 1.71–1.58 (m, 4H). ^13^C-NMR (CDCl_3_, 100 MHz): δ 119.5, 66.7, 57.5, 53.5, 25.1, 23.2, 16.9.

### 3.3. Preparation of 3,6-di(morpholin-4-y)alkyl-s-tetrazines, L3 and L4

Hydrazine (50% aqueous solution, 5.0 mL, 101 mmol) was added dropwise to a mixture of 3-(morpholin-4-yl)butanenenitrile, **4** (1.052 g, 6.8 mmol) or 3-(morpholin-4-yl)pentanenenitrile, **5** (1.148 g, 6.8 mmol), and N-acetyl-L-cysteine (1.124 g, 6.9 mmol). The reaction mixture was stirred at room temperature for 72 h under an argon atmosphere and then extracted with dichloromethane (2 × 15 mL). The organic layer was dried with MgSO_4_ and evaporated to dryness to afford a solid that was re-dissolved in dichloromethane and stirred for 96 h under an air atmosphere. During this period, the reaction crude turns progressively pink-red as the oxidation of the dihydro-*s*-tetrazine intermediate into the fully aromatic *s*-tetrazine product proceeds. After solvent evaporation, the resulting pink-red solid was suspended in hexane and filtered to remove insoluble impurities. The filtrate was evaporated to dryness to yield 3, 6-bis(morpholin-4-ylpropyl)-1,2,4,5-tetrazine (L3) or 3,6-bis(morpholin-4-ylbutyl)-1,2,4,5-tetrazine (L4). L3 was obtained in a 40% yield, as a red solid. Mp: 65 °C. IR (neat, ATR, Figure S1): ν/cm^−1^ 2959, 2928, 2854, 2755, 1460, 1363, 1111, 915. UV-vis [H_2_O, pH = 7; λmax, nm (ε, M^−1^ cm^−1^)]: 519 (522), 275 (3798). ^1^H-NMR (CDCl_3_, 400 MHz, Figure S2): δ 3.58 (t, *J* = 4.3 Hz, 8H), 3.40 (t, *J* = 7.0 Hz, 4H), 2.52 (t, *J* = 7.0 Hz, 4H), 2.52–2.40 (m, 8H), 2.16 (quin, *J* = 7.0 Hz, 4 H). ^13^C-NMR (CDCl_3_, 100 MHz, Figure S3): δ 169.9, 66.7, 58.8, 53.2, 33.1, 24.7. HRMS (ESI/Q-TOF, Figure S4) *m/z*: [M + H]^+^ Calcd. for C_16_H_29_N_6_O_2_ 337.2347; Found 337.2346; [M + 2H]^2+^ Calcd. for C_16_H_30_N_6_O_2_ 169.1210; Found 169.1210. L4 was obtained in a 42% yield, as a red solid foam. IR (neat, ATR, Figure S5): ν/cm^−1^ 2958, 2866, 2814, 2767, 1463, 1312, 914. UV−vis [H_2_O, pH = 7; λmax, nm (ε, M^−1^ cm^−1^)]: 519 (524), 279 (3758).^1^H-NMR (CDCl_3_, 400 MHz, Figure S6): δ 3.69 (t, *J* = 4.6 Hz, 8H), 3.50 (t, *J* = 7.6 Hz, 4H), 2.52–2.45 (m, 12H), 2.00 (quin, *J* = 7.6 Hz, 4H), 1.68 (q, *J* = 7.6 Hz, 4H). ^13^C-NMR (CDCl_3_, 100 MHz, Figure S7): δ 169.9, 66.5, 58.3, 53.5, 34.5, 25.9, 25.6. HRMS (EI). HRMS (ESI/Q-TOF, Figure S8) *m/z*: [M + H]^+^ Calcd. for C_18_H_33_N_6_O_2_ 365.2660; Found 365.2659; [M + Na]^+^ Calcd. for C_18_H_32_N_6_O_2_Na 387.2479; Found 387.2466.

### 3.4. Potentiometric Measurements

Potentiometric (pH-metric) titrations employed for the determination of equilibrium constants were carried out in degassed aqueous solutions at 298.1 ± 0.1 K, with a 0.1 M ionic strength, by using previously described equipment and procedures [42]. The determined ionic product of water was p*K*_w_ = 13.83 (1) (298.1 ± 0.1 K, 0.10 M Me_4_NCl). Ligand concentration was about 5 × 10^−4^ M, while anion concentration was about 2.5 × 10^−3^ M; only in the case of the L4/ClO_4_^−^ system, the anion concentration was increased to 1 × 10^−2^. The ionic strength was adjusted to 0.10 M by the addition of Me_4_NCl. The studied pH range was 2.5–11. The computer program HYPERQUAD [24] was used to calculate equilibrium constants from potentiometric data deriving from three independent titration experiments for each system. It was not possible to determine equilibrium constants in the L4/PF_6_^−^ system because of precipitation occurring at a high PF_6_^−^ concentration.

### 3.5. Isothermal Titration Calorimetry

Ligand (L3, L4) protonation and OH^−^ complexation enthalpies were determined in 0.10 M Me_4_NCl aqueous solutions at 298.1 K by using previously described equipment and procedures [5]. In a typical experiment, a NMe_4_OH solution (0.10 M, addition volumes 15 μL) was added to acidic solutions of the ligands (5 × 10^−3^ M, 1.5 cm^3^). Three titrations were performed for each ligand. The computer program HypΔH [43] was used to calculate reaction enthalpies from calorimetric data. Corrections for the heats of dilution were applied.

### 3.6. X-ray Structure Analyses

Pink crystals of L3 (a), L4 (b), H_2_L3(ClO_4_)_2_∙2H_2_O (c), H_2_L4(ClO_4_)_2_∙2H_2_O (d), H_2_L3(PF_6_)_2_ (e), and H_2_L3(PF_6_)_2_∙2H_2_O (f) were used for X-ray diffraction analysis. A summary of the crystallographic data is reported in Table 2. The integrated intensities were corrected for Lorentz and polarization effects and an empirical absorption correction was applied [44]. The structures were solved by direct methods (SIR-92) [45]. Refinements were performed by means of full-matrix least-squares using SHELXL Version 2014/7 [46]. All the non-hydrogen atoms were anisotropically refined.

Hydrogen atoms were usually introduced in a calculated position and their coordinates were refined according to the linked atoms, with the exception of the acidic protons of H_2_L3(ClO_4_)_2_∙2H_2_O (c), H_2_L3(PF_6_)_2_ (e), and H_2_L3(PF_6_)_2_∙2H_2_O (f). Hydrogen atoms belonging to the cocrystallized water molecules were localized in the Fourier density maps, and freely refined. The isotropic displacement parameters for the two water hydrogens were set to be equal by using EADP instruction. He hexafuorophosphate ion in the asymmetric unit of H_2_L3(PF_6_)_2_ (e) was found to be spread over two different positions and the fluorine atoms were freely anisotropically refined with an o.f.s equal to 0.46 and 0.54. CCDC 1853188–1853193 contain the crystallographic data for these structures.

### 3.7. Computational Studies

The conformation of the adducts formed by L1–L4 and their diprotonated species (H_2_L^2+^) with a water molecule were obtained in the gas phase by means of density functional theory (DFT) calculations. The starting coordinates for each input structure were built by using the crystallographic coordinates of the proper diprotonated complex (L1: boat conformation in H_2_L1(ClO_4_)_2_∙2H_2_O, planar conformation in H_2_L1(PF_6_)_2_∙2H_2_O [8]; L2: chair conformation in H_2_L2Cl_2_ [9], planar conformation in H_2_L2(PF_6_)_2_∙H_2_O [8]; L3: chair conformation in H_2_L1(ClO_4_)_2_∙2H_2_O [8], planar conformation in H_2_L3(PF_6_)_2_; L4: H_2_L4(ClO_4_)_2_∙2H_2_O). After removing all atoms not pertaining to the diprotonated ligands, the water molecule was manually placed over the tetrazine ring for each adduct, except for [L4(H_2_O)] in planar conformation where the H-bonded water molecule is in the original crystallographic position. In the case of the neutral adducts (unprotonated ligands), the acidic protons on the morpholine nitrogen atoms were also removed. Each adduct was fully minimized with Jaguar [47] at the DFT/M06-2X level of theory, 6-311G**+ basis set. The nature of stationary points as true minima was checked by frequency calculations.

## 4. Conclusions

The anion binding ability of the *s*-tetrazine-based L1–L4 ligands is preserved even when the ligand side chains become longer and the contribution to complex stability due to hydrogen bonding with side chain functionalities extinguishes. Indeed, thermodynamic data for the formation of ClO_4_^−^ and PF_6_^−^ complexes are consistent with a major contribution from anion–π interactions to complex stability and with a participation of hydrogen bonds with side chains (protonated or not) that decreases with chain length. In the solid state, the prominence of anion–π interactions is clearly observed. Indeed, the crystal structures of H_2_L3(ClO_4_)_2_∙2H_2_O, H_2_L3(PF_6_)_2_, H_2_L3(PF_6_)_2_∙2H_2_O, and H_2_L4(ClO_4_)_2_∙2H_2_O complexes invariably show the anions located above the tetrazine ring at close anion–π contact distance, while the positively charged (protonated) morpholine nitrogen atoms of the side chains are not in contact with the anions and only weak hydrogen bonds with hydrogen atoms of the aliphatic chains are intermittently found. Accordingly, the tetrazine ring appears to be a valuable element for the construction of anion receptors and, beyond them, for the construction of receptors for all kind of substrates carrying lone pairs of electrons.

A notable characteristic of these ligands is the aptitude of the neutral (not-protonated) molecules to bind OH^−^ in water to form complexes of high stability, relative to complexes with other anions. As far as we know, L1–L4 are the first organic receptors that have so far been reported as capable of binding OH^−^ anions in water. Dissection of the free energy changes of complexation into enthalpic and entropic contributions shows different behaviours of L1 and L2 relative to L3 and L4. In the case of L1 and L2, the largely favorable enthalpic terms (ΔH° < 0) are counterbalanced by unfavorable entropy changes (TΔS° < 0), while in the case of L3 and L4, the OH^−^ binding reactions are less exothermic (less favorable) but the entropic contributions become favorable (TΔS° > 0), a behaviour that can be ascribed to a different involvement of the ligand side chains in OH^−^ binding, which is stronger for the shorter ligands (L1 and L2) and weaker (or absent) for the longer ones (L3 and L4).

## Figures and Tables

**Table 1:** Thermodynamic parameters for ligand protonation and anion complex formation determined at 298.1 ± 0.1 K in 0.10 M Me_4_NCl aqueous solution. Values in parentheses are the standard deviation on the last significant figure. Δ*G*°, Δ*H*° and *T*Δ*S*° values in kJ/mol.

		L1	L2	L3	L4
L + H^+^ = HL^+^	−log*K*	4.45 (3) ^a^	6.19 (1) ^a^	7.18 (3) ^c^	7.59 (6) ^c^
	Δ*G*°	−25.4 (2) ^a^	−35.32 (6) ^a^	−41.0 (2)	−43.3 (4)
	Δ*H*°	−4.6 (4) ^a^	−26.8 (4) ^a^	−41.6 (2)	−39.6 (1)
	*T*Δ*S*°	20.8 (4) ^a^	8.5 (4) ^a^	−0.6 (4)	3.7 (5)
HL^+^ + H^+^ = H_2_L^2+^	log*K*	3.45 (3) ^a^	5.37 (1) ^a^	6.54 (3) ^c^	6.84 (6) ^c^
	Δ*G*°	−19.7 (2) ^a^	−30.64 (6) ^a^	−37.3 (2) ^c^	−39.0 (4) ^c^
	Δ*H*°	4.6 (4) ^a^	−18.4 (4) ^a^	−47.9 (2) ^c^	−39.7 (1) ^c^
	*T*Δ*S*°	24.3 (4) ^a^	12.2 (4) ^a^	−10.6 (4) ^c^	−0.7 (5) ^c^
L + OH^−^ = [(L)OH]^−^	log*K*	2.60 (7) ^b^	1.76 (7) ^b^	2.69 (3) ^c^	2.40 (6) ^c^
	Δ*G*°	−14.8 (4) ^b^	−10.0 (4) ^b^	−15.3 (2) ^c^	−13.7 (3) ^c^
	Δ*H*°	−25.1 (4) ^b^	−18.8 (4) ^b^	−9.6 (2) ^c^	−9.5 (1) ^c^
	*T*Δ*S*°	−10.3 (8) ^b^	−8.8 (8) ^b^	5.7 (4) ^c^	4.2 (4) ^c^
L + ClO_4_^−^ = [(L)ClO_4_]^−^	log*K*		1.98 (5) ^a^	2.26 (9) ^c^	1.1 (1) ^c^
HL^+^ + ClO_4_^−^ = [(HL)ClO_4_]	log*K*	2.07 (9) ^a^	2.26 (5) ^a^	2.2 (1) ^c^	1.1 (2) ^c^
H_2_L^2+^ + ClO_4_^−^ = [(H_2_L)ClO_4_]^+^	log*K*	2.31 (8) ^a^	2.51 (4) ^a^	2.1 (1) ^c^	1.6 (1) ^c^
L + PF_6_^−^ = [(L)PF_6_]^−^	log*K*	1.96 (8) ^a^	3.07 (8) ^a^	2.30 (9) ^c^	
HL^+^ + PF_6_^−^ = [(HL)PF_6_]	log*K*	2.67 (7) ^a^	3.17 (7) ^a^	2.2 (1) ^c^	
H_2_L^2+^ + PF_6_^−^ = [(H_2_L)PF_6_]^+^	log*K*	2.98 (7) ^a^	3.39 (8) ^a^	2.4 (1) ^c^	

^a^ Taken from [5]. ^b^ Taken from [6]. ^c^ This work.

**Table 2:** Crystal data and structure refinement for L3 (a), L4 (b), H_2_L3(ClO_4_)_2_∙2H_2_O (c), H_2_L4(ClO_4_)_2_∙2H_2_O (d), H_2_L3(PF_6_)_2_ (e), and H_2_L3(PF_6_)_2_∙2H_2_O (f).

	(a)	(b)	(c)	(d)	(e)	(f)
Empirical formula	C_16_H_28_N_6_O_2_	C_18_H_32_N_6_O_2_	C_16_H_34_Cl_2_N_6_O_12_	C_18_H_38_Cl_2_N_6_O_12_	C_16_H_30_F_12_N_6_O_2_P_2_	C_16_H_34_F_12_N_6_O_4_P_2_
Formula weight	336.44	364.50	573.39	601.44	628.40	664.43
Temperature (K)	150	298	150	150	150	150
space group	P -1	P 2_1_/c	P 2_1_/c	P 2_1_/c	P 2_1_/n	P 2_1_/c
a (Å)	6.6983 (6)	13.5805 (3)	13.7608 (5)	14.4546 (3)	7.7907 (3)	13.4690 (4)
b (Å)	8.6859 (8)	5.1482 (1)	7.3316 (3)	17.0531 (4)	8.8430 (3)	7.8954 (2)
c (Å)	8.7604 (9)	14.2994 (3)	13.3194 (7)	11.0345 (2)	18.2505 (8)	13.6108 (4)
α (°)	109.119 (9)	90	90	90	90	90
β (°)	95.224 (8)	104.627 (2)	111.655 (5)	97.932 (2)	96.454 (4)	112.191 (4)
γ (°)	109.872 (8)	90	90	90	90	90
Volume (Å^3^)	440.99 (8)	967.34 (4)	1248.94 (10)	2693.93 (10)	1249.37 (8)	1340.20 (7)
Z	1	2	2	4	2	2
Independent reflections/R(int)	1619/0.0454	1849/0.0271	2125/0.0837	4991/0.0693	6350/0.0333	2458/0.0895
μ (mm^−1^)	0.704 (Cu-Kα)	0.679 (Cu-Kα)	2.983 (Cu-Kα)	2.793 (Cu-Kα)	0.292 (Mo-Kα)	2.604 (Cu-Kα)
R indices [I>2σ(I)] ^a^	R1 = 0.0698	R1 = 0.0416	R1 = 0.0801	R1 = 0.0599	R1 = 0.0410	R1 = 0.0548
	wR2 = 0.1995	wR2 = 0.1077	wR2 = 0.2447	wR2 = 0.1513	wR2 = 0.1017	wR2 = 0.0964
R indices (all data) ^a^	R1 = 0.1012	R1 = 0.0504	R1 = 0.1409	R1 = 0.1006	R1 = 0.0634	R1 = 0.1162
	wR2 = 0.2168	wR2 = 0.1152	wR2 = 0.3190	wR2 = 0.1917	wR2 = 0.1161	wR2 = 0.1217

^a^ R1 = Σ || Fo| − |Fc||/Σ |Fo|; wR2 = [ Σ w (Fo^2^ − Fc^2^) ^2^/Σ wF.

## Supplementary Materials

The following are available online. Selected contacts in crystal structures; ^1^H and ^13^C-NMR, FTIR, and HRMS (Q-TOF/ESI) spectra of L3 and L4; ^1^H-NMR spectra of ClO_4_^−^ and PF_6_^−^ complexes with L3 and L4; correlations of structural data; fittings for the first and the second series of protonation constants; calculated complex conformations. Crystallographic information files are available from the CCDC, reference numbers 1853188–1853193.
