# Supplementary material for: Solid State and Solution Study on the Formation of Inorganic Anion Complexes with a Series of Tetrazine-Based Ligands
Source: Molecules. 2019 Jun 16;24(12):2247. doi: 10.3390/molecules24122247 (PMC6631435; doi:10.3390/molecules24122247)
Supplement: Supplementary file 1 [file molecules-24-02247-s001.pdf]

# Supplementary Materials

## Thermodynamic and Structural Study on the Formation of Inorganic Anion Complexes with a Series of Tetrazine-Based Ligands

Matteo Savastano,[a] Celeste García-Gallarín,[b] Claudia Giorgi,[a] Paola Gratteri,[c] Maria Dolores López de la Torre,[b] Carla Bazzicalupi,[a*] Antonio Bianchi,*[a] Manuel Melguizo*[b]

[a] Department of Chemistry "Ugo Schiff", University of Florence, Via della Lastruccia 3, 50019, Sesto Fiorentino, Italy.

[b] Department of Inorganic and Organic Chemistry, University of Jaén 23071, Jaén, Spain.

[c] Department NEUROFARBA – Pharmaceutical and nutraceutical section; Laboratory of Molecular Modeling Cheminformatics & QSAR, University of Firenze, via Ugo Schiff 6, 50019 Sesto Fiorentino, Italy.

Table S1. Selected contacts (Å) in the $H_2L3(ClO_4)_2 \cdot 2H_2O$ crystal structure. Esd in parentheses.

| | |
|---|---|
| N1–C4 | 3.414(9) |
| O3–C5 | 3.36(1) |
| C8–O6 | 3.08(1) |
| C3–O5 | 3.306(8) |
| C6–O4 | 3.561(8) |
| O2–O3 | 2.843(7) |
| O1–O2 | 2.830(7) |
| H1–O2 | 1.98(7) |

Table S2. Selected contacts (Å) in the $H_2L4(ClO_4)_2 \cdot 2H_2O$ crystal structure. Esd in parentheses.

| | |
|---|---|
| N5–O4 | 2.766(4) |
| O4–O11 | 2.832(4) |
| O4–O14 | 2.814(4) |
| O12–C15 | 3.171(4) |
| O12–C16 | 3.255(4) |
| O14–C8 | 3.208(5) |
| O13–C15 | 3.177(5) |
| O13–C2 | 3.387(5) |
| O14–C9 | 3.218(4) |
| C17–O23 | 3.172(4) |
| O3–C9 | 3.197(4) |
| O23–O3 | 2.939(4) |
| O22–C6 | 3.245(4) |
| O21–O3 | 2.847(4) |
| N6–O3 | 2.721(4) |

Table S3. Selected contacts (Å) in the $H_2L3(PF_6)_2$ crystal structure. Esd in parentheses.

| | |
|---|---|
| C3–F1B/F1A | 3.284(9)/3.32(1) |
| C6–F3B/F3A | 3.37(1)/3.37(1) |
| C8–F3B/F3A | 3.29(1)/3.39(1) |
| C7–F5B/F5A | 3.05(1)/2.95(1) |
| C5–F5B/F5A | 3.17(1)/3.09(1) |
| C3–F6B/F6A | 3.172(7)/3.129(.008) |
| C8–F6B/F6A | 3.266(8)/3.297(.009) |

Table S4. Selected contacts (Å) in the $H_2L3(PF_6)_2 \cdot 2H_2O$ crystal structure. Esd in parentheses.

| | |
|---|---|
| C5–F2 | 3.308(5) |
| C8–F1 | 3.153(5) |
| C8–.F6 | 3.162(5) |
| C4–F5 | 3.157(5) |
| C6–F3 | 3.269(5) |

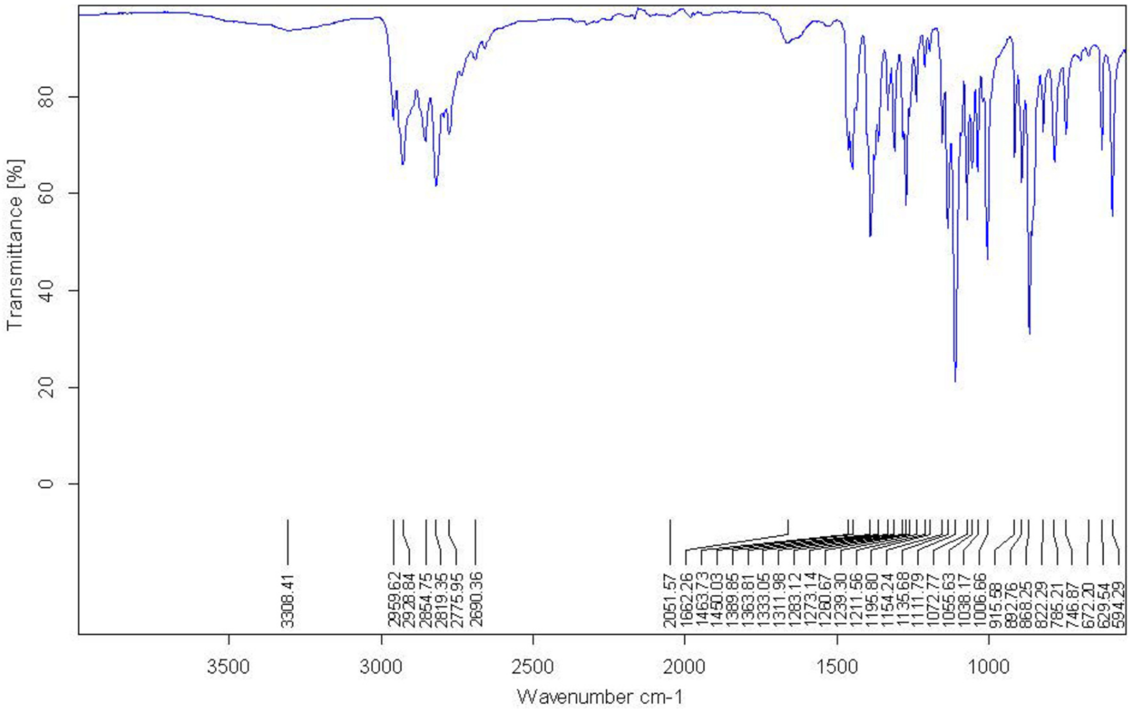


Figure S1. FTIR (ATR) of L3.

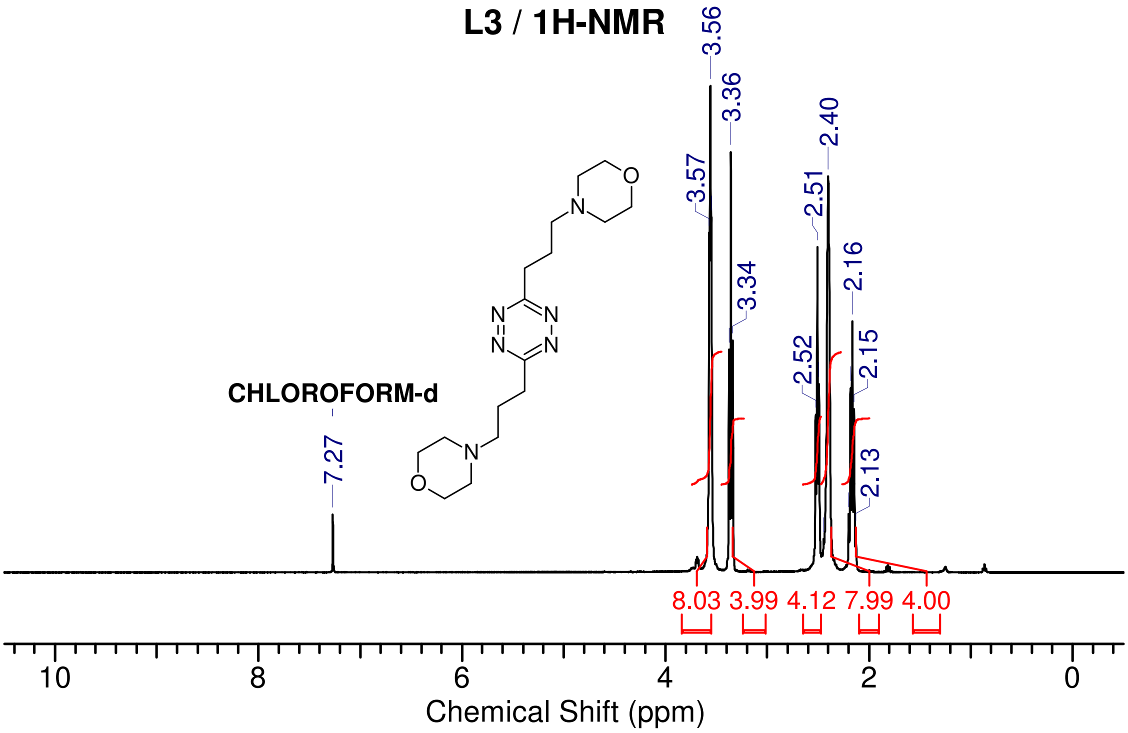


Figure S2. $^{1}$H-NMR ($CDCl_3$, 400 MHz) spectrum of L3.

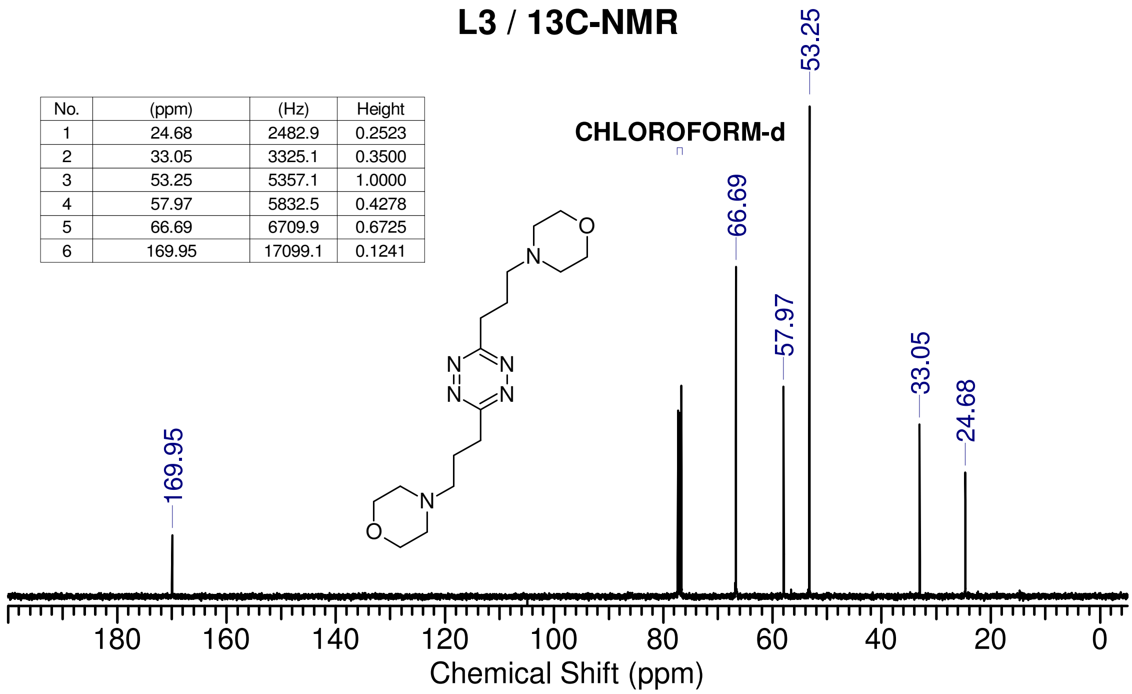

| No. | (ppm) | (Hz) | Height |
| --- | --- | --- | --- |
| 1 | 24.68 | 2482.9 | 0.2523 |
| 2 | 33.05 | 3325.1 | 0.3500 |
| 3 | 53.25 | 5357.1 | 1.0000 |
| 4 | 57.97 | 5832.5 | 0.4278 |
| 5 | 66.69 | 6709.9 | 0.6725 |
| 6 | 169.95 | 17099.1 | 0.1241 |



Figure S3. $^{13}$C-NMR ($CDCl_3$, 100 MHz) spectrum of L3.

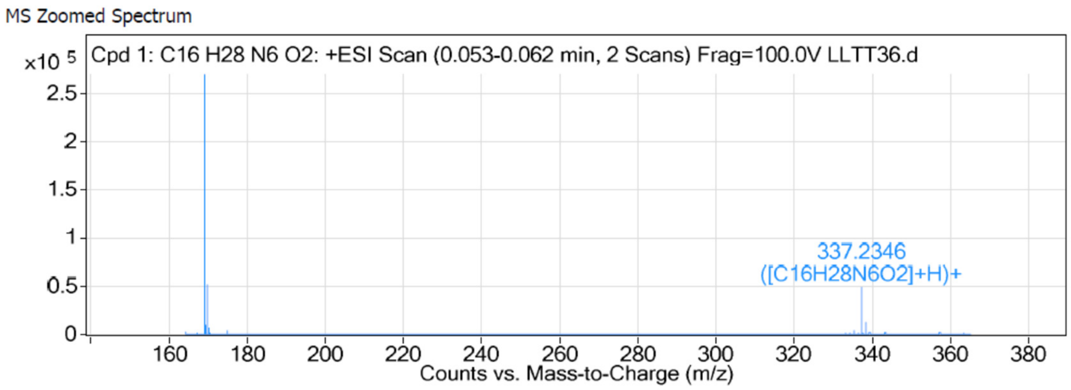


Figure S4. HRMS (Q-TOF/ESI) spectrum of L3.

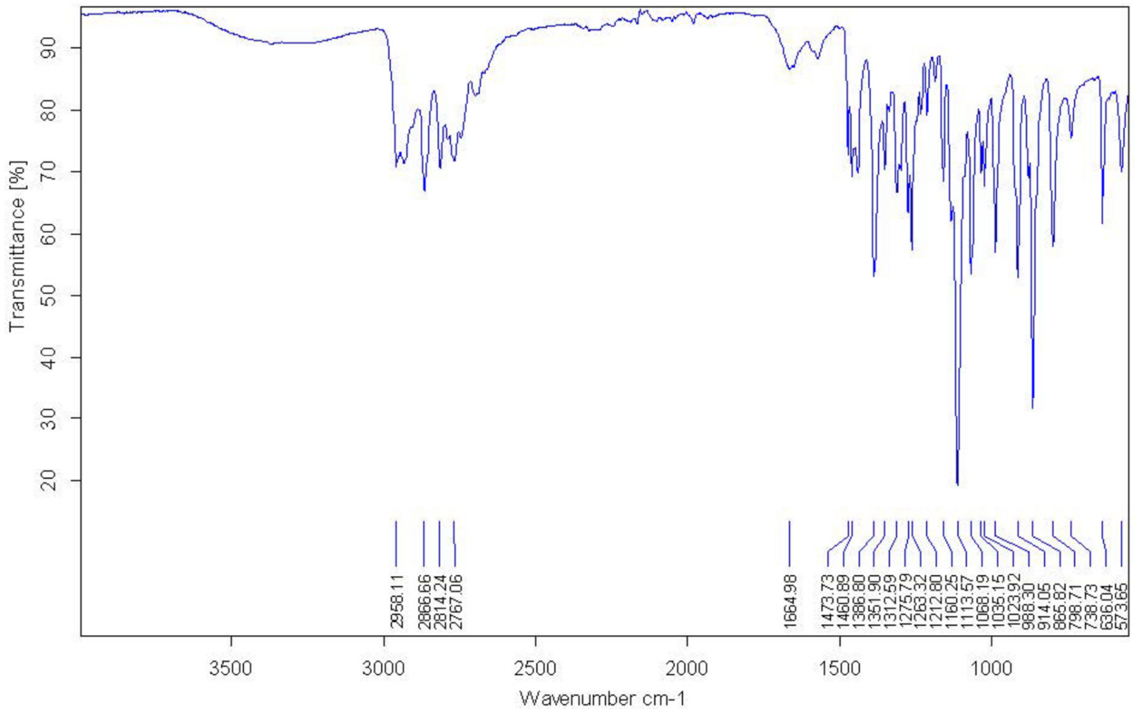


Figure S5. FTIR (ATR) of L4.

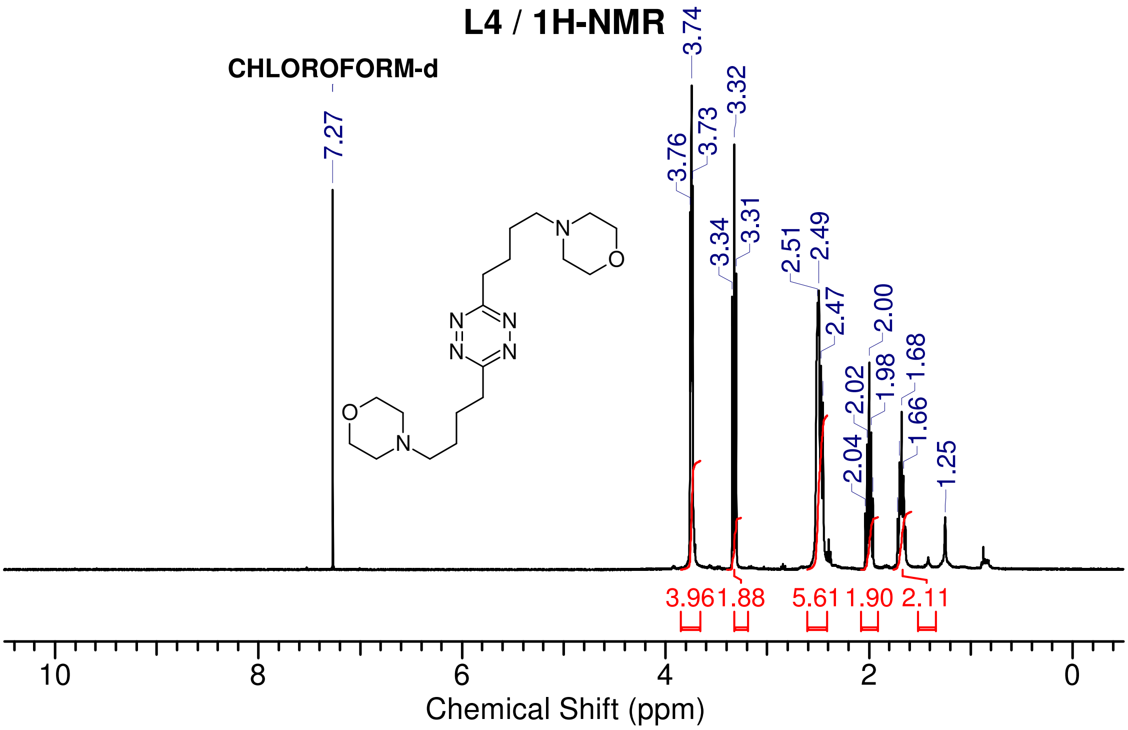


Figure S6. $^{1}$H-NMR ($CDCl_3$, 400 MHz) spectrum of L4.

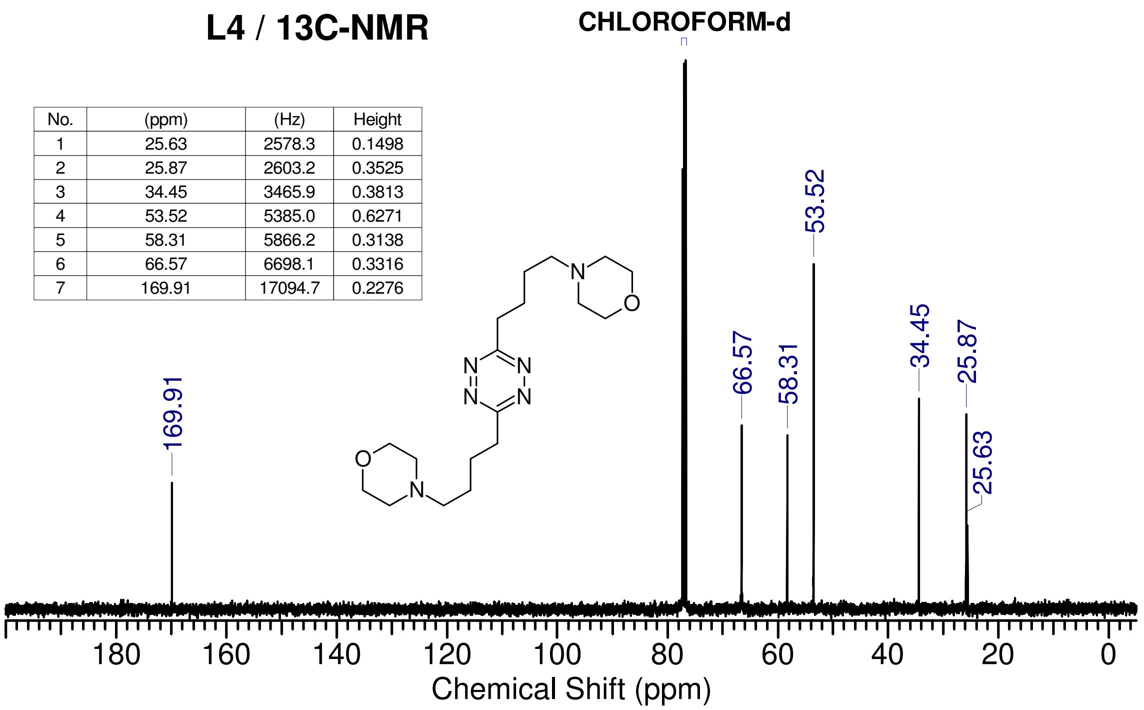


| No. | (ppm) | (Hz) | Height |
|---|---|---|---|
| 1 | 25.63 | 2578.3 | 0.1498 |
| 2 | 25.87 | 2603.2 | 0.3525 |
| 3 | 34.45 | 3465.9 | 0.3813 |
| 4 | 53.52 | 5385.0 | 0.6271 |
| 5 | 58.31 | 5866.2 | 0.3138 |
| 6 | 66.57 | 6698.1 | 0.3316 |
| 7 | 169.91 | 17094.7 | 0.2276 |

Figure S7. $^{13}$C-NMR ($CDCl_3$, 100 MHz) spectrum of L4.

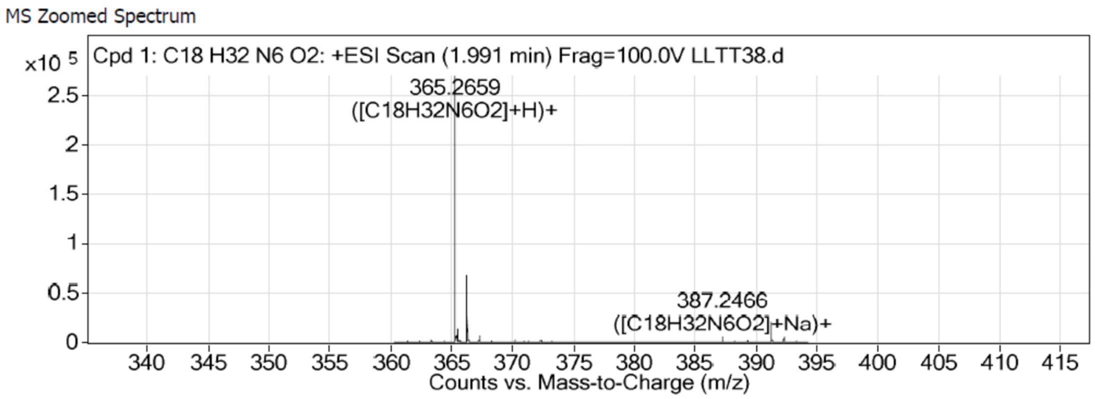


Figure S8. HRMS (Q-TOF/ESI) spectrum of L4.

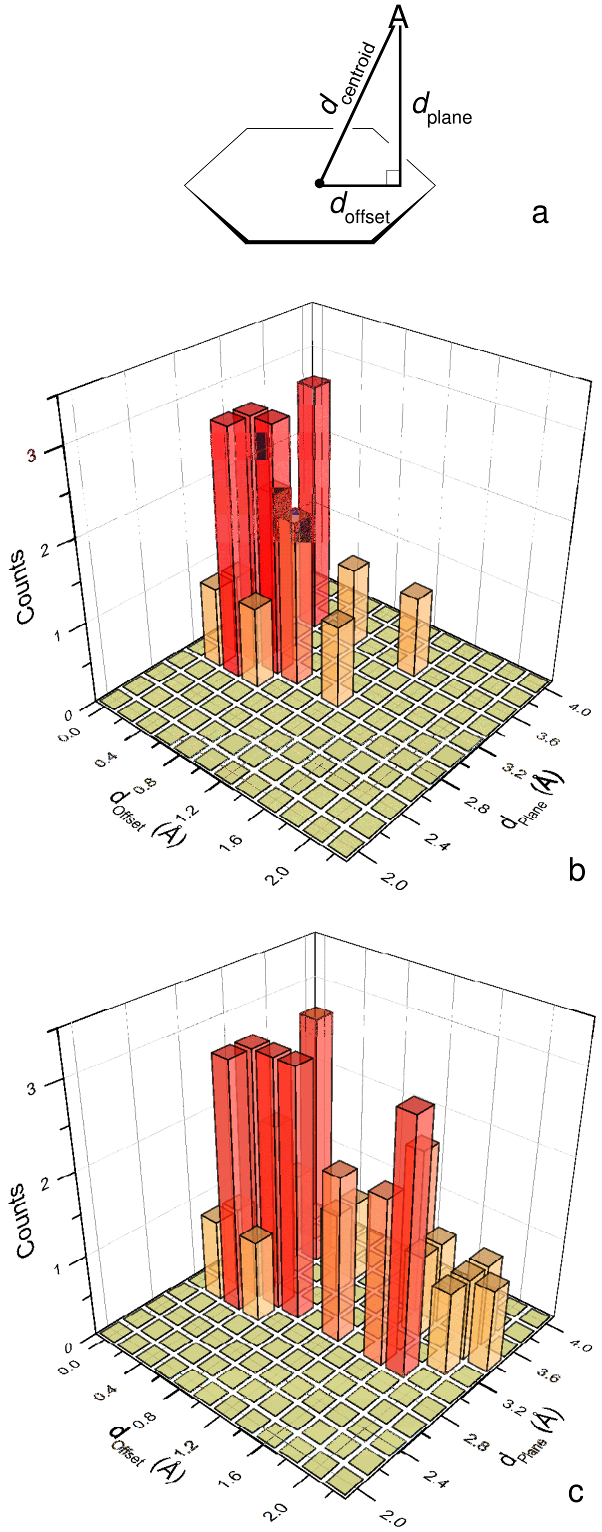


Figure S9. (a) Displacement of an atom A from the centre (centroid) of the tetrazine ring. (b) Frequency of shorter anion-ring contacts, for each anion, in the $d_{plane}$ *vs* $d_{offset}$ plane. (c) Frequency of all anion-ring contacts in the $d_{plane}$ *vs* $d_{offset}$ plane.

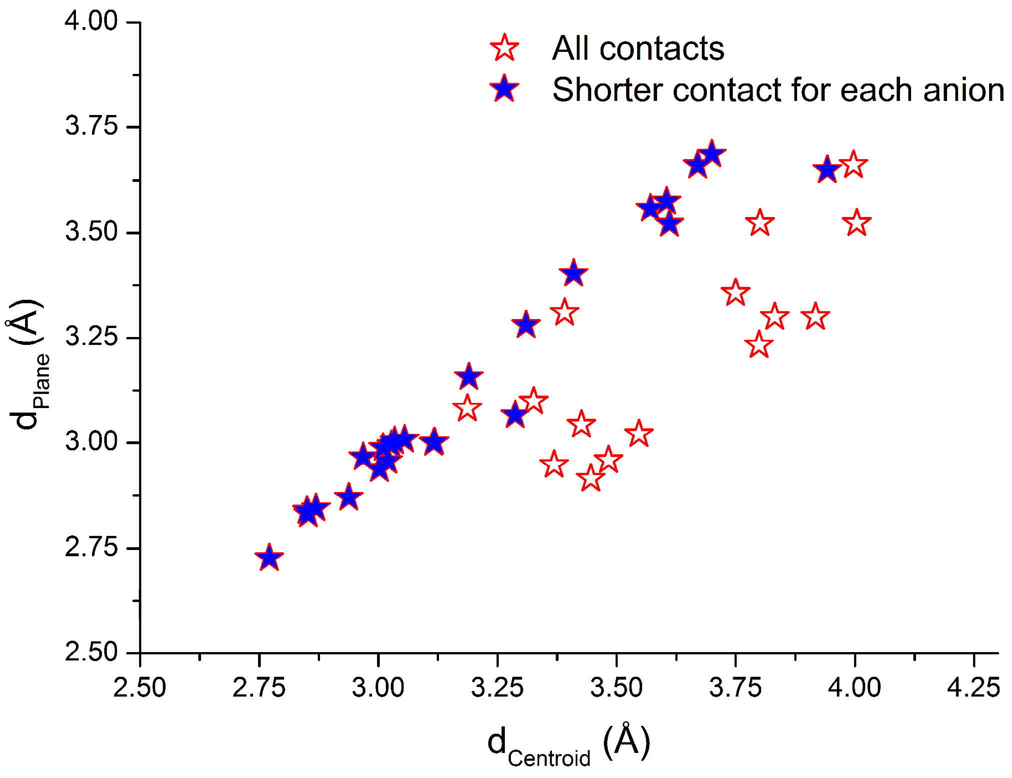


Figure S10. Correlation between $d_{plane}$ and $d_{centroid}$ distances in the crystal structures of L1-L4 anion complexes.

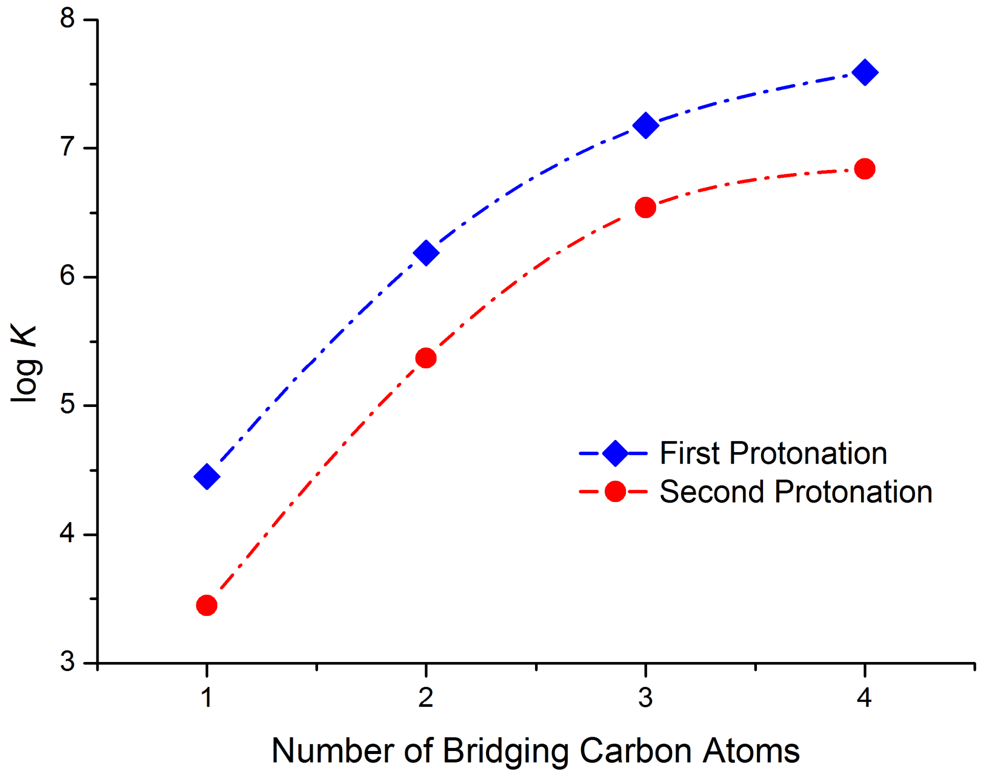


Figure S11. Hyperbolic fitting for the first and the second series of protonation constants (log*K*) of L1-L4 ligands.

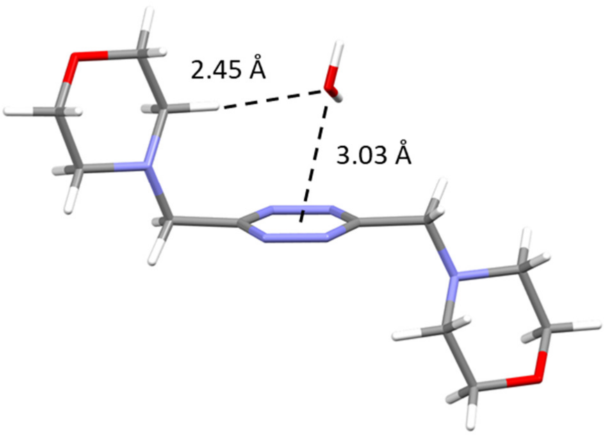


Figure S12. Calculated conformation for the [L1($H_2O$)] adduct.

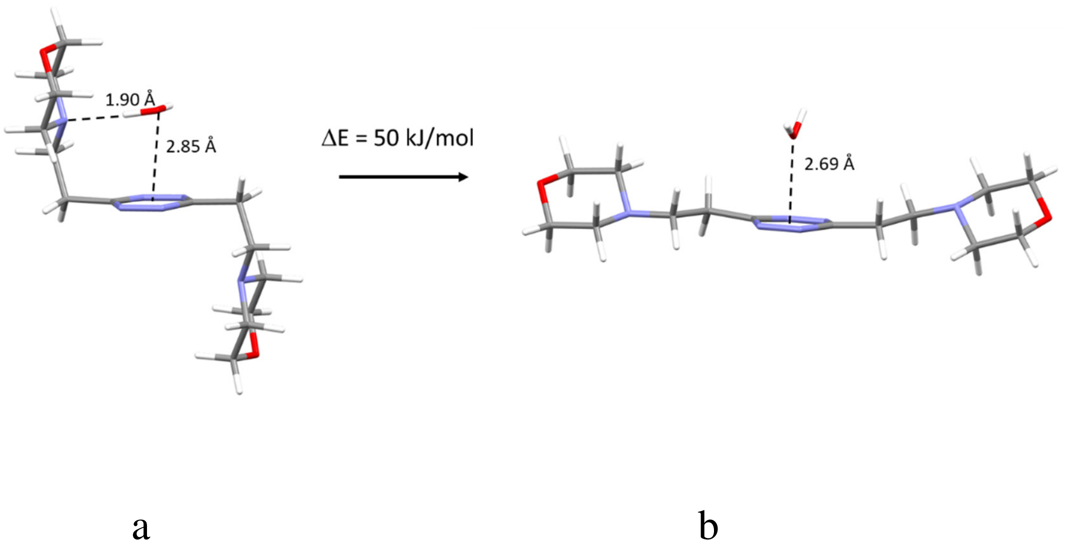


Figure S13. Calculated conformations for the [L2($H_2O$)] adduct: a) chair conformation, b) planar conformation.

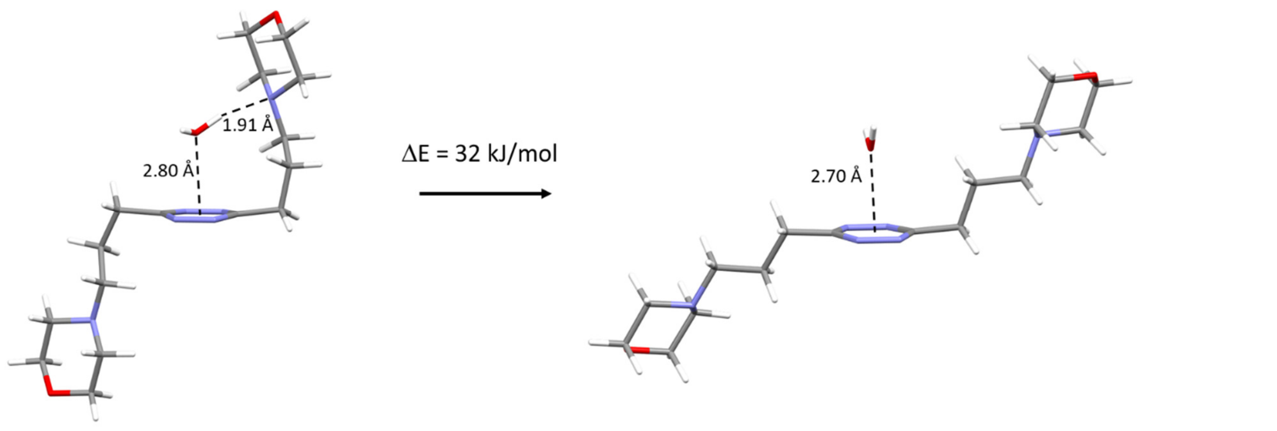


a b

Figure S14. Calculated conformations for the [L3($H_2O$)] adduct: a) chair conformation, b) planar conformation.

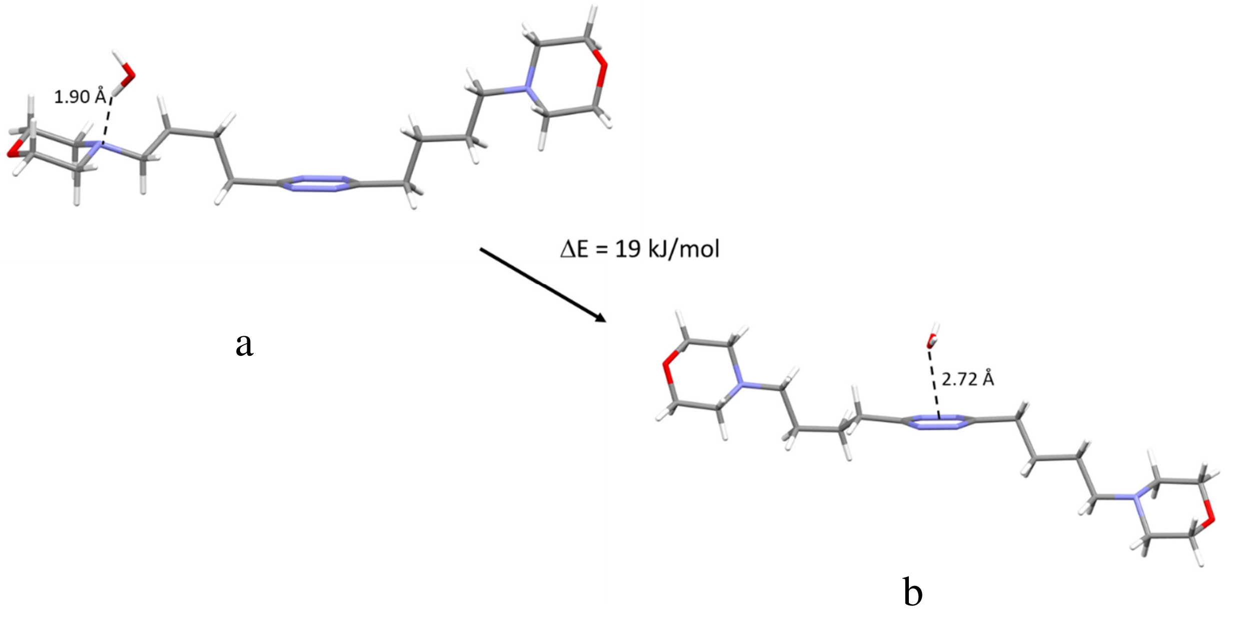


Figure S15. Calculated conformations for the [L4($H_2O$)] adduct: a) planar conformation, H-bond; b) planar conformation, lone pair-π.

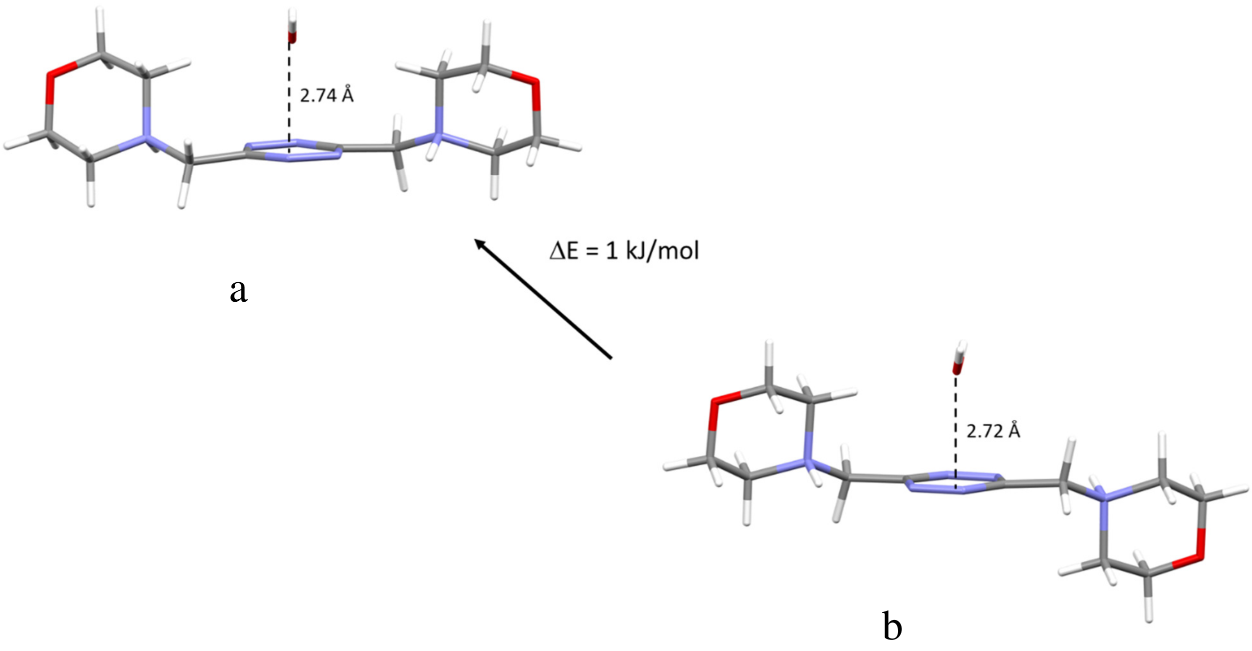


Figure S16. Calculated conformations for the $[H_2L1(H_2O)]^{2+}$ adduct: a) boat conformation, b) chair conformation.

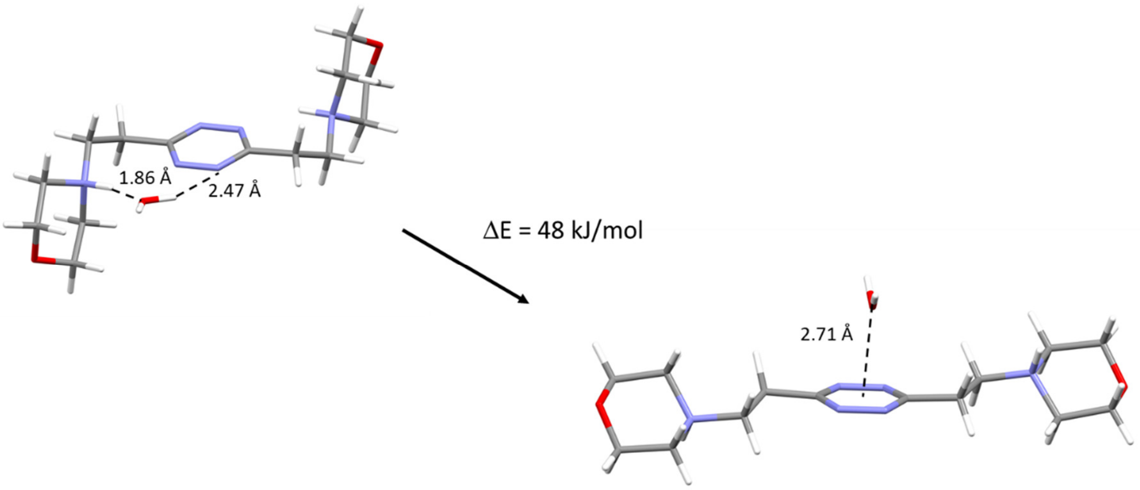


Figure S17. Calculated conformations for the $[H_2L2(H_2O)]^{2+}$ adduct: a) chair conformation, b) planar conformation.

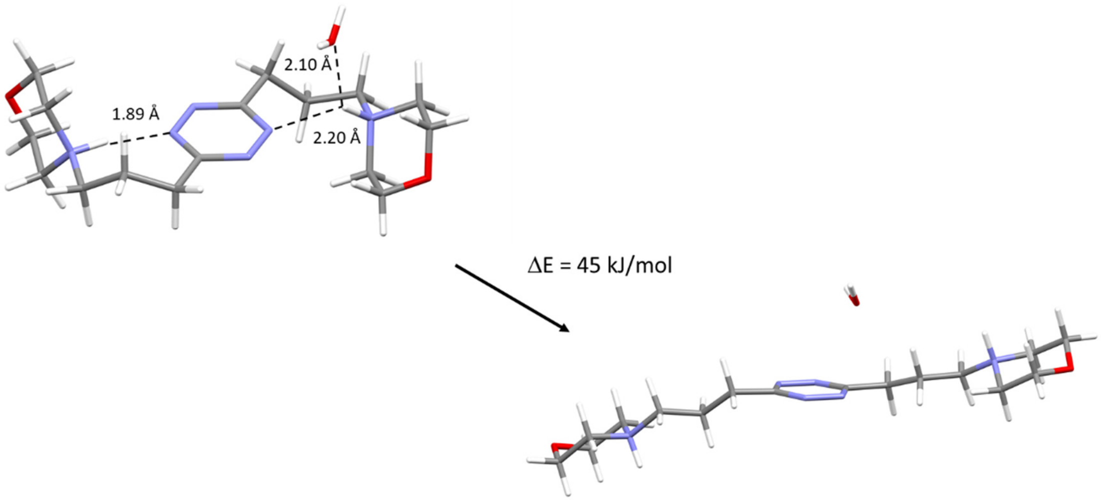


Figure S18. Calculated conformations for the $[H_2L3(H_2O)]^{2+}$ adduct.

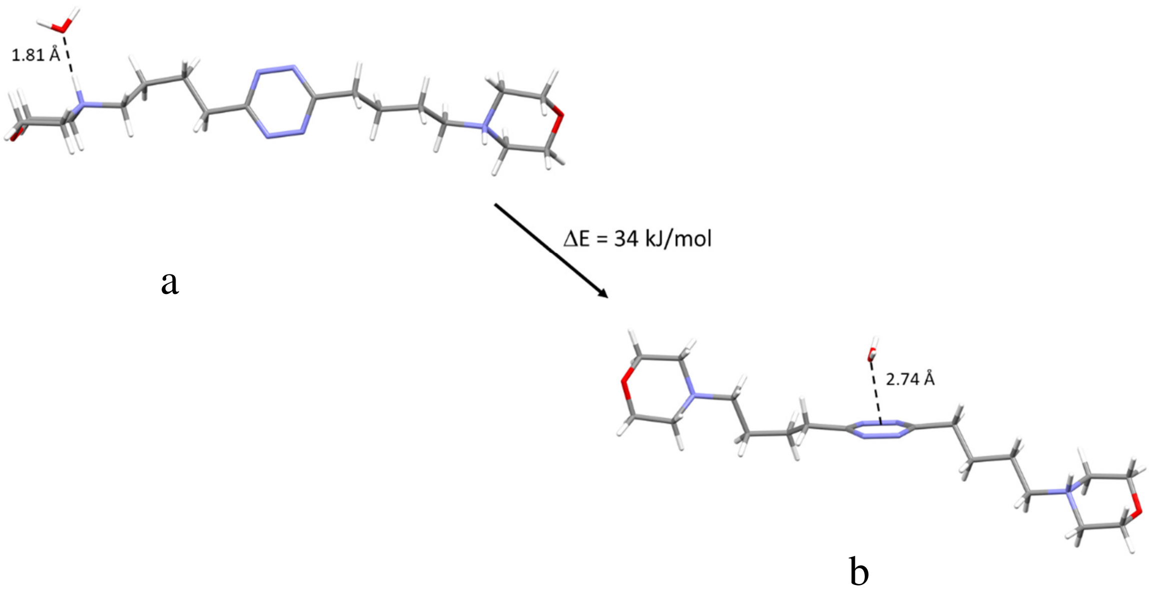


Figure S19. Calculated conformation for the $[H_2L4(H_2O)]^{2+}$ adduct: a) planar conformation, H-bond; b) planar conformation, lone pair-π.

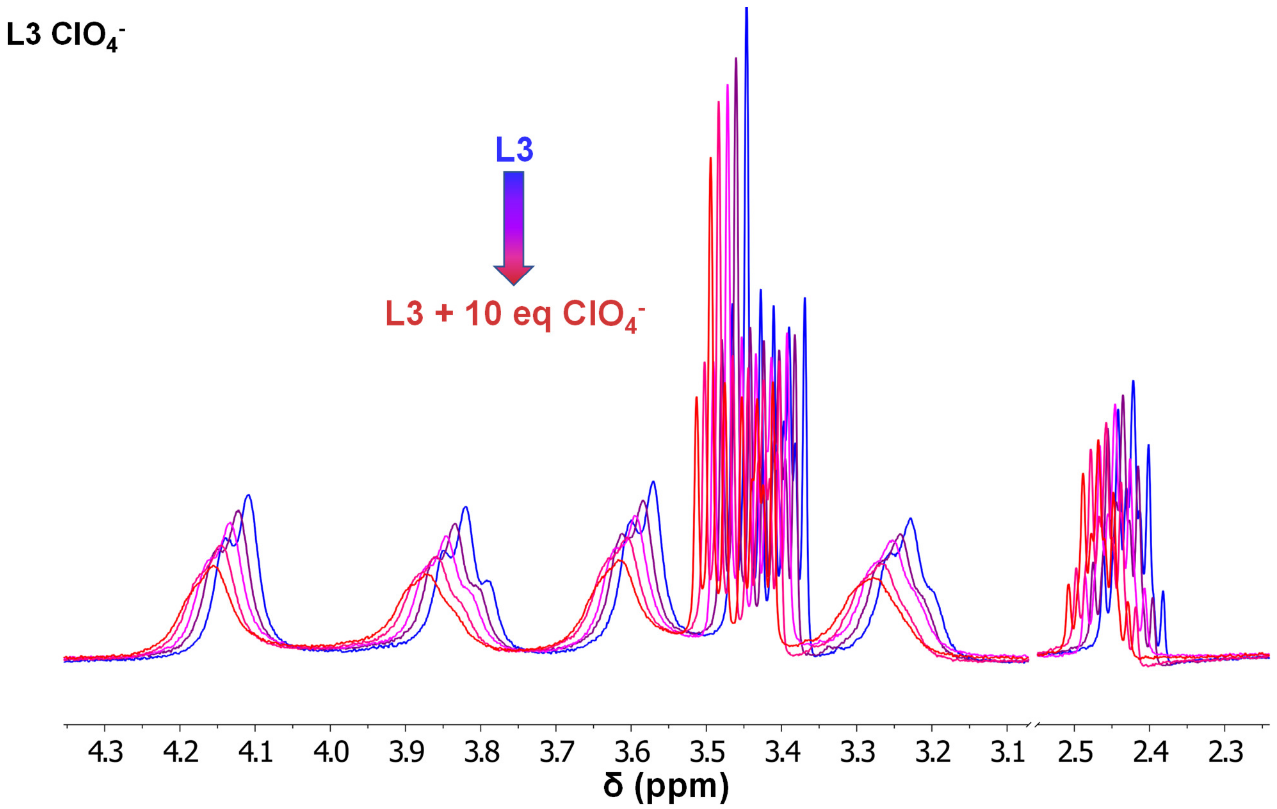


Figure S20. $^{1}$H NMR spectra of L3 recorded in $D_2O$ (pH 4) in the absence and in the presence of increasing amounts of $ClO_4^-$.

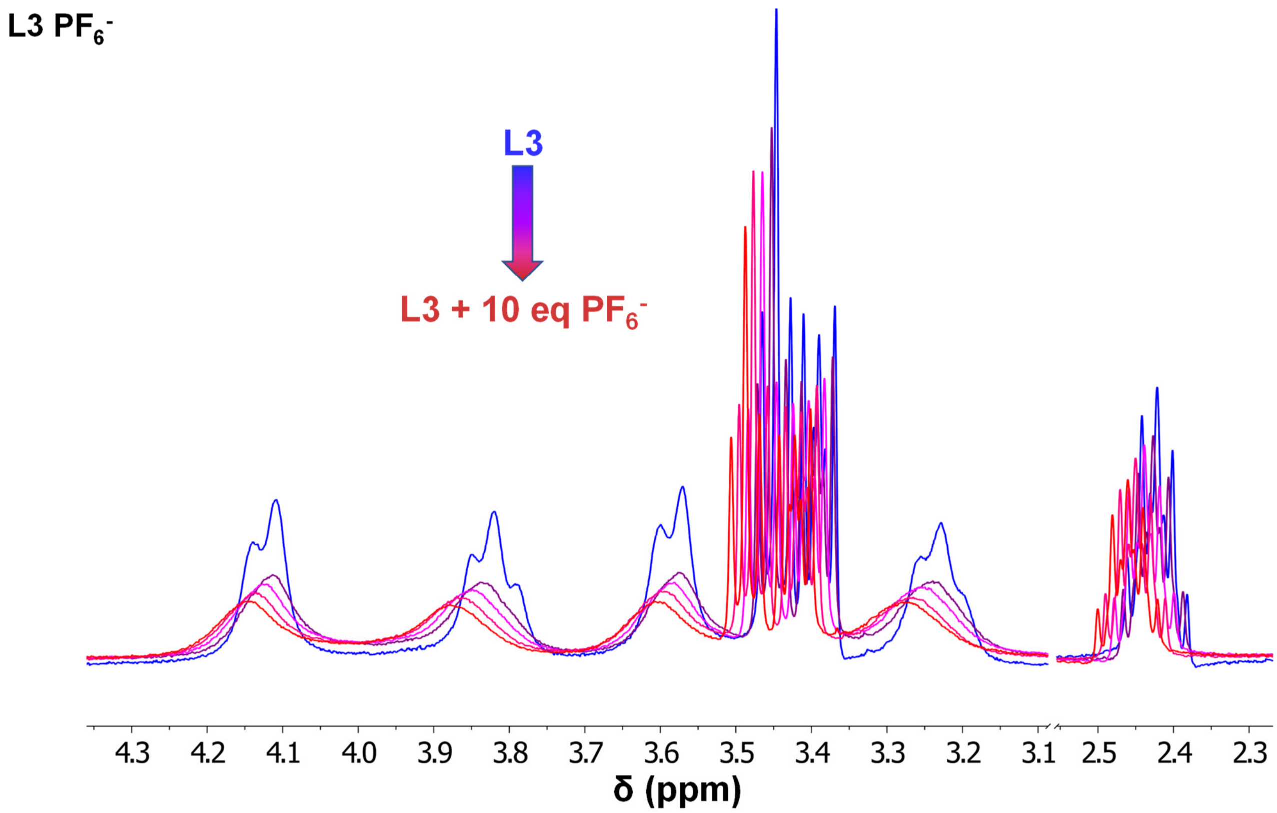


Figure S21. $^{1}$H NMR spectra of L3 recorded in $D_2O$ (pH 4) ) in the absence and in the presence of increasing amounts of $PF_6^-$.

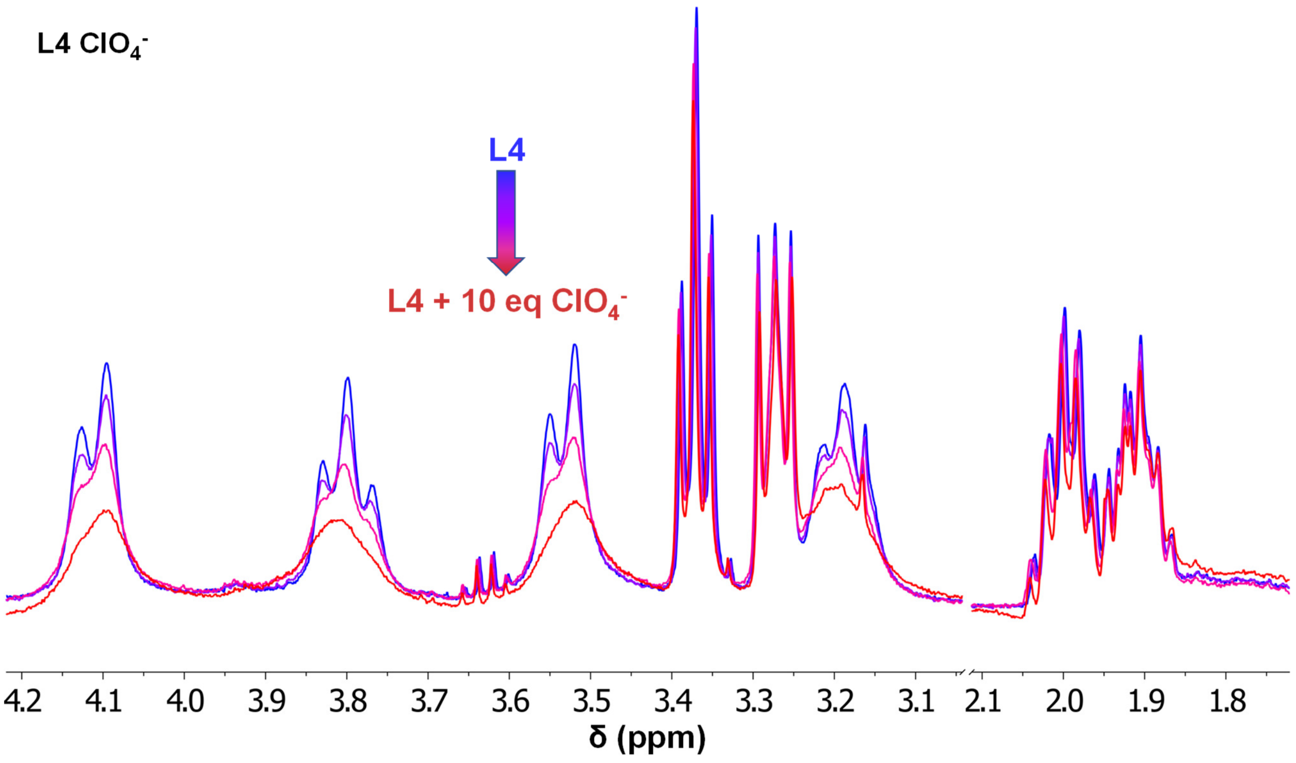


Figure S22. $^{1}$H NMR spectra of L4 recorded in $D_2O$ (pH 4) in the absence and in the presence of increasing amounts of $ClO_4^-$.

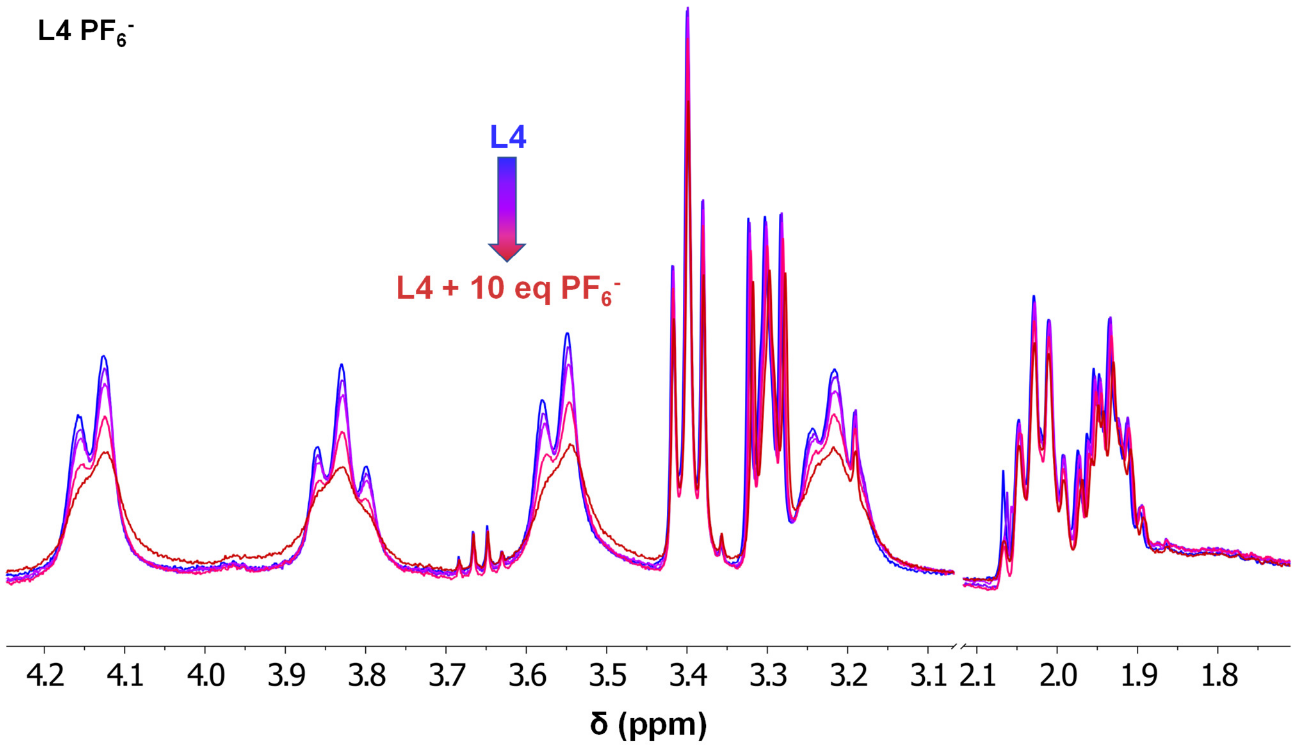


Figure S23. $^{1}$H NMR spectra of L4 recorded in $D_2O$ (pH 4) ) in the absence and in the presence of increasing amounts of $PF_6^-$.
